# An inflammatory gene signature distinguishes neurofibroma Schwann cells and macrophages from cells in the normal peripheral nervous system

**DOI:** 10.1038/srep43315

**Published:** 2017-03-03

**Authors:** Kwangmin Choi, Kakajan Komurov, Jonathan S. Fletcher, Edwin Jousma, Jose A. Cancelas, Jianqiang Wu, Nancy Ratner

**Affiliations:** 1 Division of Experimental Hematology and Cancer Biology, Cancer and Blood Diseases Institute, Cincinnati Children’s Hospital Medical Center, Department of Pediatrics, University of Cincinnati, Cincinnati, OH 45229, USA; 2 Hoxworth Blood Center, College of Medicine, University of Cincinnati, Cincinnati, OH 45229, USA

## Abstract

Neurofibromas are benign peripheral nerve tumors driven by *NF1* loss in Schwann cells (SCs). Macrophages are abundant in neurofibromas, and macrophage targeted interventions may have therapeutic potential in these tumors. We generated gene expression data from fluorescence-activated cell sorted (FACS) SCs and macrophages from wild-type and mutant nerve and neurofibroma to identify candidate pathways involved in SC-macrophage cross-talk. While in 1-month-old *Nf1* mutant nerve neither SCs nor macrophages significantly differed from their normal counterparts, both macrophages and SCs showed significantly altered cytokine gene expression in neurofibromas. Computationally reconstructed SC-macrophage molecular networks were enriched for inflammation-associated pathways. We verified that neurofibroma SC conditioned medium contains macrophage chemo-attractants including colony stimulation factor 1 (CSF1). Network analysis confirmed previously implicated pathways and predict novel paracrine and autocrine loops involving cytokines, chemokines, and growth factors. Network analysis also predicted a central role for decreased type-I interferon signaling. We validated type-I interferon expression in neurofibroma by protein profiling, and show that treatment of neurofibroma-bearing mice with polyethylene glycolyated (PEGylated) type-I interferon-α2b reduces the expression of many cytokines overexpressed in neurofibroma. These studies reveal numerous potential targetable interactions between *Nf1* mutant SCs and macrophages for further analyses.

Neurofibromatosis type 1 (NF1) is one of the most common human monogenic disorders, affecting about 0.3% of the human population. Nearly half of NF1 patients develop plexiform neurofibromas, a benign peripheral nerve sheath tumor associated with significant patient morbidity. Human neurofibromas contain Schwann cells (SCs) with biallelic *NF1* mutation^1^. In mice, biallelic loss of *Nf1* in the SC lineage results in plexiform neurofibroma formation^2^,^3^. In human and mouse, biallelic *NF1* mutation/loss causes loss of function of neurofibromin protein, with no evidence of dominant negative or gain of function effects^4^.

*NF1* encodes neurofibromin, an off-signal for RAS proteins. Active, Guanosine-5′-triphosphate (GTP)-bound RAS is therefore present in higher levels in *NF1* mutant cells than in normal cells, particularly after cell stimulation^4^. RAS-GTP has been implicated in inflammation; RAS-GTP expression increased transcription of *IL8/CXCL8*, which initiated inflammation in a xenograft model^5^. Pro-inflammatory cytokine signaling can cooperate with RAS pathway hyper-activation to drive malignant tumor development^6^,^7^,^8^. Few systems that allow for the analysis of benign tumor formation over time have been used to study inflammatory processes.

Current evidence suggests that an inflammatory environment is critical for neurofibroma development and growth. Loss of *Nf1* enhances inflammatory gene expression in cultured SCs^9^, and injury-associated inflammation facilitates neurofibroma development in mouse models^10^,^11^,^12^. Mast cells are present in both human and mouse neurofibromas and are necessary for tumor development in some mouse models^13^. We recently found that Iba1^+^/F4/80^+^/CD11b^+^ macrophages comprise 20–40% of neurofibroma cells in mouse and human neurofibromas^14^. In the *Nf1*^*fl/fl*^*;DhhCre* plexiform neurofibroma model, the *DhhCre* driver effects *Nf1* loss in SCs at embryonic day 12.5, with about 50% of SCs showing *Nf1* loss^3^. All mice develop nerve hyperplasia with macrophage recruitment and visible benign neurofibromas by 4 months of age; tumors begin to compress the spinal cord by 7 months of age. Transformation to malignancy does not occur. In the *Nf1*^*fl/fl*^*;DhhCre* plexiform neurofibroma model, pharmacological inhibition of macrophage/mast cell function with a dual Kit/Fms (c-kit/Csf1r) kinase inhibitor reduced macrophage accumulation and growth of established neurofibromas (age 7–9 months)^14^. Thus, macrophages in established neurofibromas may contribute to neurofibroma growth.

Here, we verify that the *Nf1* gene is wild-type in macrophages in the *Nf1*^*fl/fl*^*;DhhCre* mouse model. Therefore, this genetically engineered mouse (GEM) neurofibroma model allows monitoring of changes downstream of *Nf1* loss/elevated RAS-GTP specifically in SCs, over time, in a predictable model of benign neurofibroma formation. These changes in SCs may affect tumor macrophages that are wild-type at *Nf1*. We posited that identifying differentially expressed genes (DEGs) that comprise the pro-tumorigenic SC-macrophage interactome would provide clues to aid in development of macrophage-targeted anti-neurofibroma therapies. We characterized the SC-macrophage signaling network at both early (initiation) and late (neurofibroma) time points using microarray gene expression. We validated secretion of macrophage chemoattractant(s) by neurofibroma SC, utilized computational multicellular gene network reconstruction to identify central genes and target pathways in the SC-macrophage interactome, and validated this analysis by targeting CSF1 *in vitro* and interferon signaling *in vivo*. The data provide numerous avenues for future study.

## Results

### Neurofibromas contain SCs and macrophages

**Figure 1:**
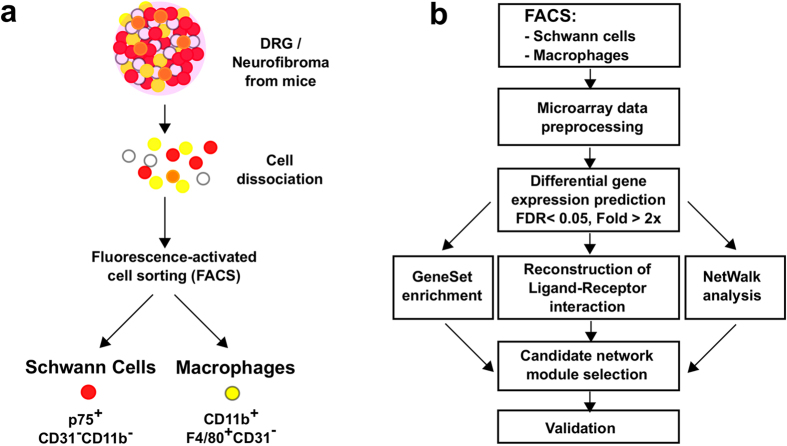
Overall analysis pipeline. (**a**) DRG and neurofibroma tumors were dissociated and sorted into SC and macrophage populations. (**b**) DEGs were detected in comparisons of 7- to 1-month-old cell populations. These DEG lists were used to run gene set enrichment analysis and to reconstruct a ligand-receptor interaction map. Combined with NetWalk analysis, we narrowed down our target gene lists by identifying the most relevant gene network modules in neurofibroma. Cytokine arrays were used to validate the differential protein level changes of several target genes (between wild-type DRG and neurofibroma tumors).

We collected dorsal root ganglia (DRG), nerve roots, and associated brachial plexus and sciatic nerve from 1-month-old *Nf1*^*fl/fl*^*;DhhCre* mice and *Nf1*^*fl/fl*^ controls. At 1 month of age, neurofibromas have not yet formed but nerve development is complete. Thus, SC differentiation (myelination and formation of Remak bundles) has occurred, and SC basal lamina is present^15^. For 1-month-old mice, we pooled tissues from 3–4 mice for each of three FACS analysis. We collected neurofibromas, which grow to encompass these nerve structures, from 7-month-old *Nf1*^*fl/fl*^*;DhhCre* mice for comparison. Content of F4/80^+^;CD11b^+^ macrophages was 2.6 fold (P < 0.01) higher in 7-month-old *Nf1*^*fl/fl*^*;DhhCre* (0.77 ± 0.09%) than in 1-month-old *Nf1*^*fl/fl*^*;DhhCre* mice (0.27 ± 0.03%) or 1-month-old *Nf1*^*fl/fl*^*mice* (0.23 ± 0.03%) (Supplementary Fig. S1). We FACS-sorted p75^+^ SCs from mice expressing a green fluorescent protein (GFP) reporter allele (*Nf1*^*fl/fl*^; *DhhCre;b-actin* lox-stop-lox enhanced green fluorescent protein (EGFP)). In these mice, EGFP serves as a marker of Cre-mediated recombination. We found that 51.5% of p75^+^ cells are p75^+^/EGFP^+^, and 42.9% are p75^+^/EGFP^−^, confirming prior descriptions of this model (not shown)^3^. We sorted F4/80^+^;CD11b^+^ macrophages in three independent experiments (Fig. 1a). We used the sorted cells to perform microarray experiments and analyze the data according to the analysis pipeline summarized in Fig. 1b.

### Characterization of sorted SCs and macrophages

To verify that the p75^+^ SCs (sorting marker) overlap with Dhh-Cre mediated recombination in SCs but not macrophages, we analyzed tumors from *DhhCre;Nf1fl/fl;b-actin* lox-stop-lox EGFP mice. In these mice, as noted above, EGFP serves as a marker of Cre-mediated recombination. We genotyped FACS sorted p75^+^;EGFP^+^ and p75^+^;EGFP^−^ SCs. p75^+^;EGFP^+^ SCs showed expected Cre-mediated recombination of *Nf1*. p75^+^;EGFP^−^ SCs cells showed recombination in about 25% of alleles, implying that some of these cells also recombine *Nf1*, but do not recombine β-actin to drive EGFP expression (Supplementary Fig. S2a). Therefore, cells analyzed from *Nf1*^*fl/fl*^*;DhhCre* mice are a mixture of wild-type and *Nf*^*-−/−*^ SCs. FACS sorted F4/80^+^;CD11b^+^ macrophages were *Nf1*^*fl/fl*^ wild-type (Supplementary Fig. S2b).

To further validate the identities of the sorted populations, we generated heatmaps from the gene expression data for panels of known SC and macrophage markers. Strongly supporting that the sorted cells are bona fide SCs and macrophages, sorted p75^+^ SC samples display high expression of established SC marker genes (n = 27) and low expression of macrophage marker genes (n = 12); the opposite pattern of expression was seen for F4/80^+^;CD11b^+^ sorted macrophages (Supplementary Fig. S3a,b).

### Both neurofibroma SCs and macrophages actively modulate inflammatory gene expression

**Figure 2:**
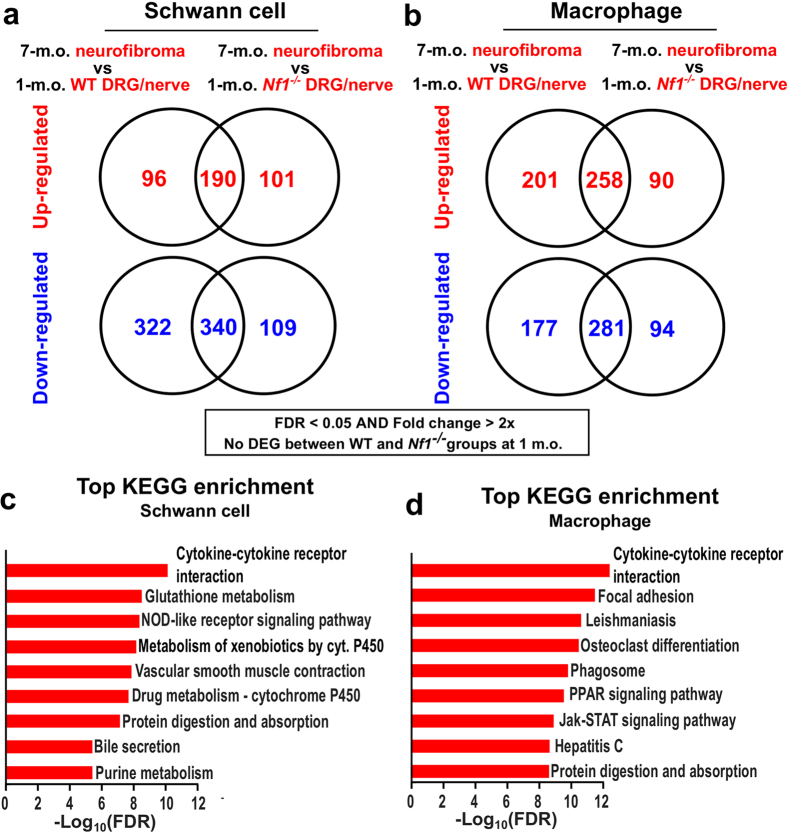
DEGs and gene set enrichment analysis. DEGs were predicted in (**a**) 7-to-1 month SC comparison and (**b**) 7-to-1-month-old macrophage comparison, using the limma method (fold change >2x and FDR q < 0.05). KEGG pathway analyses were performed using WegGestalt webserver using DEGs from (**c**) 7(*Nf1*^*−/−*^)-to-1(*Nf1*^*−/−*^) month SC comparison and (**d**) 7(*Nf1*^+*/*+^)-to-1(*Nf1*^+*/*+^) month neurofibroma macrophages. The designation *Nf1*^*−/−*^ represents SCs from *Nf1*^*fl/fl*^;*DhhCre* mice; a mixture of wild-type and *Nf1*^*−/−*^ SCs.

The *DhhCre* driver causes *Nf1* loss in SCs during embryogenesis^3^. Nevertheless, using fold change >2x and false discovery rate (FDR) q < 0.05 as criteria, no significant DEGs were detected in comparisons of SCs and macrophages from 1-month-old *Nf1*^*fl/fl*^*;DhhCre* nerves to 1-month-old *Nf1*^*fl/fl*^ (wild-type) nerve/DRG SCs and macrophages. We then compared 7-month-old *Nf1*^*fl/fl*^*;DhhCre* (neurofibroma) SCs to either 1-month-old *Nf1*^*fl/fl*^*;DhhCre* nerve SCs or 1-month-old *Nf1*^*fl/fl*^ (wild-type) nerve/DRG SCs to identify DEGs, using the same filtering criteria (Fig. 2a). Macrophages (7-month-old neurofibroma *Nf1*^*fl/fl*^*;DhhCre* to both 1-month-old groups) were also compared using the same criteria (Fig. 2b). DEGs were detected in both comparisons.

Kyoto Encyclopedia of Genes and Genomes (KEGG) pathway enrichment analyses on the DEGs reported “Cytokine-cytokine receptor interaction” as the most enriched pathway in both SCs (Fig. 2c) and macrophages (Fig. 2d). Additional inflammation-associated pathways were also highly ranked, suggesting that an inflammatory microenvironment distinguishes neurofibromas from normal peripheral nerves.

### Neurofibroma macrophages express both M1 and M2 signature genes

**Figure 3:**
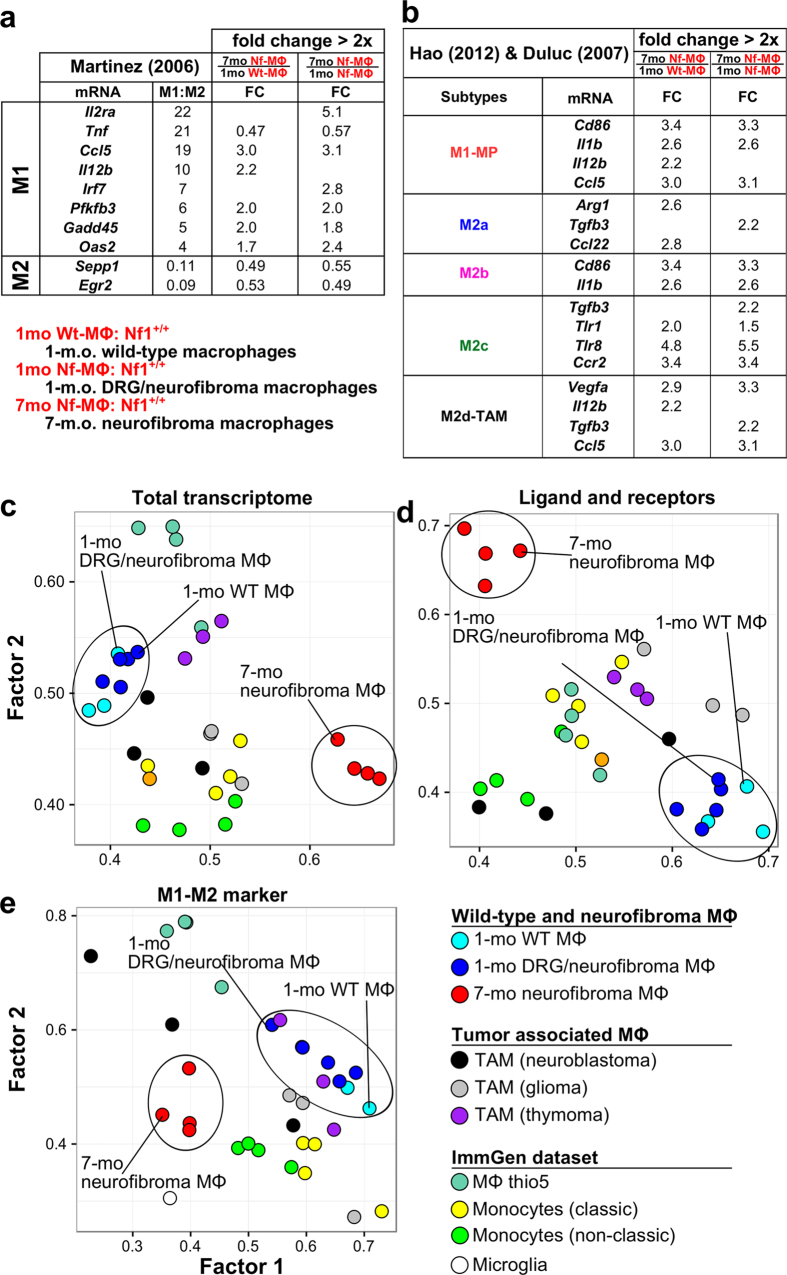
Characteristics of 7 neurofibroma macrophages. DEGs from 7-to-1 month comparison of macrophages (**a**,**b**) were mapped to M1/M2 polarization signature genes collected from previous publications. Only differentially expressed signature genes were displayed. Macrophage (MΦ) subpopulation clusters were generated by exploratory factor analysis (EFA) approach, based on (**c**) all genes in the microarray, (**d**) ligands and receptor genes, and (**e**) M1/M2 signature genes^19^.

The M1/M2 polarization theory was derived from analysis of the response of macrophages to bacteria and pathogens^16^,^17^ and provides a framework commonly used to describe macrophages in tumors. Although this construct has limitations, in general, tumor-associated macrophages (TAMs) shift their gene expression patterns from a pro-inflammatory M1-like gene expression profile toward an anti-inflammatory M2-like profile^18^. To characterize 7-month-old neurofibroma macrophages (*Nf1* wild-type), we mapped the DEGs from 7-to-1 month comparisons to M1/M2 polarization signature genes collected from published studies^19^,^20^,^21^,^22^ (Fig. 3a and b). Interestingly, 7-month-old neurofibroma macrophages differentially expressed many typical M1-like signature genes and did not clearly fall into M2 sub-populations, suggesting that benign neurofibroma macrophages express an admixture of M1 and M2 genes.

### Neurofibroma macrophage expression profiles are distinct from other relevant macrophage sub-populations

In tumors, macrophages can be derived from local normal tissue and/or recruited from bone marrow monocytes that differentiate into macrophages in the tumor microenvironment. Nerve-resident macrophages, monocytes, inflammatory macrophages, and/or TAMs might be present in neurofibromas. To better characterize the cells, we compared neurofibroma macrophages with normal macrophage/monocyte subgroups (GSE37448) from the Immunological Genome Project (ImmGen, https://www.immgen.org/) and published TAM datasets, including glioma, neuroblastoma, and thymoma TAMs (GSE59047). To visualize the relatedness among sample types, we carried out exploratory factor analysis (EFA)^23^ on gene expression profiles from total DEGs (Fig. 3c), differentially expressed ligand-receptor genes (Fig. 3d), and differentially expressed M1/M2 polarization signature genes (Fig. 3e)^19^,^20^. In these analyses, 7-month-old neurofibroma macrophages separated from 1-month-old macrophages. One-month-old macrophages from wild-type and *Nf1*^*fl/fl*^*;DhhCre* mice clustered together, consistent with our inability to identify genes showing significant differential expression between 1-month-old groups. Importantly, 7-month-old neurofibroma macrophages did not cluster together with previously defined macrophage cell populations. Dendritic cells separated significantly from all of these populations (not shown). This analysis supports the ideas that (1) peripheral nerve macrophages are a distinct cell population, and (2) neurofibroma macrophages differ from resident macrophages and alter gene expression in recruited and/or local cells.

### Neurofibroma SCs express M1/M2 signature genes

**Figure 4:**
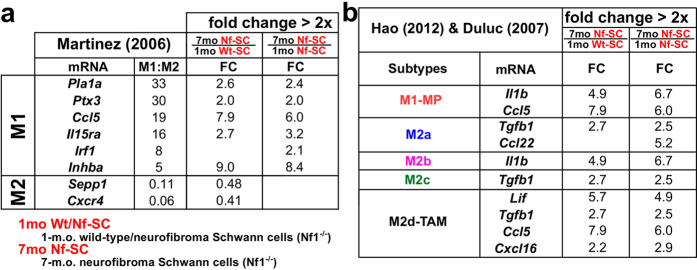
Differentially expressed M1-M2 signature genes in neurofibroma SCs. DEGs from 7-to-1 month comparison of SCs (**a**,**b**) were mapped to M1/M2 polarization signature genes collected from previous publications. Only differentially expressed signature genes are displayed.

Interestingly, 7-month-old neurofibroma SCs, like macrophages, differentially expressed several M1/M2 signature genes (Fig. 4). Consistent with known alterations in cytokine/chemokine expression and inflammatory mediators after nerve injury, this observation implies an active role of *Nf1*^*−/−*^ SCs in modulating local immune responses^24^,^25^. Two pro-inflammatory genes*, Il1b* and *Ccl5,* were up-regulated both in macrophages and SCs, and their gene expression fold changes were larger in SCs (*Il1b* (6.7x) and *Ccl5* (5.9x)) than in macrophages (*Il1b* (2.6x) and *Ccl5* (3.1x)). SCs in injured nerves secrete IL1B to initiate acute inflammation during the recovery process^26^,^27^,^28^. *Nf1*^*−/−*^ SCs may similarly initiate nerve inflammation by secreting IL1B.

### Ligand-receptor interaction map reveals potential autocrine and/or paracrine cell-cell interactions

Given that neurofibromas can be incited by wounding and tumors behave as wounds that do not heal, we sought factors (e.g. growth factors, chemokines, cytokines, interferons (types-I and -II), and/or interleukins) that might reflect an injury environment, and/or serve as recruitment factors for immune cells. Many secreted factors play critical roles in inflammation, immunosuppression, and cancer growth via autocrine and/or paracrine signaling in diverse settings^29^,^30^,^31^,^32^, but the specific factors that might act in neurofibroma are largely unknown. To visualize possible intra- and inter-cellular interaction interfaces in neurofibromas, we constructed a ligand-receptor interaction map based on well-annotated public data sources. DEGs were assigned to the map (Supplementary Fig. S4). This map predicts autocrine and paracrine regulatory units in the 7-month-old neurofibroma microenvironment.

### Chemokine family

*Ccl5 (Rantes*) is a macrophage chemoattractant^33^ and was up-regulated both in 7-month-old neurofibroma SCs (6.0x) and macrophages (3.2x). There were no transcriptional changes in its major receptor gene, *Ccr5*, but another CCL5 receptor gene, *Ccr3*, was down-regulated (0.38x). The chemokine CCL2 and its receptor CCR2 are also important for macrophage recruitment in some systems. *Ccr2* expression (3.4x) increased in macrophages (Supplementary Fig. S4).

### Interferon family

We found that expression of a type-I interferon (IFN-β) gene is down-regulated and type-II interferon (IFN-γ) gene is up-regulated, so that imbalance between type-I and type-II inteferons might be characteristic of neurofibromas. A certain level of negative feedback control between the two types of interferons has been described^34^,^35^. IFN-γ promotes pro-inflammatory responses including full activation of macrophages^36^. *Ifna14* and *Ifnb1* were down-regulated in SCs (0.45x) and macrophages (0.40x) respectively. *Ifnb1* was also slightly down-regulated both in 1-month-old *Nf1*^*−/−*^ SCs and 1-month-old *Nf*^+*/*+^ macrophages from *Nf1*^*fl/fl*^*;DhhCre* mice compared to their wild-type controls, suggesting that levels of IFN-β mRNA might be reduced even in early stages of neurofibroma growth. *Ifngr1* was up-regulated in macrophages (2.0x) while its ligand gene *Ifng* was slightly up-regulated both in SCs (1.7x) and macrophages (1.7x), suggesting possible feedback autocrine and/or paracrine signaling between type-I and type-II interferons.

### Interleukins

Interleukin 1 beta (IL1B) is activated by CASP1-mediated cleavage and plays key roles in inflammatory responses, including recruitment of macrophages^37^. *Il1b* was up-regulated both in SCs (6.7x) and macrophages (2.6x); its receptor gene (*Il1r1*) was not differentially expressed. Human plexiform neurofibroma SCs also show up-regulated *IL1B* gene expression (GSE14038), supporting the relevance of this observation.

### Other cytokines and growth factors

Up-regulation of *Kitl*^9^, *Tgfb1*^38^, and *Btc*^39^ has been described previously in *Nf1*-related tumorigenesis, and we confirmed up-regulation of mRNAs encoding these ligands in our analysis of SCs (*Kitl* (2.8x), *Tgfb1* (2.2x), and *Btc* (betacellulin, 3.3x). We also identified significant increases in genes not previously studied in neurofibroma. *Csf1* (4.4x), a macrophage differentiation factor, *Lif* (4.9x), and *Inhba* (8.4x) were up-regulated in SCs; *Vegfa* in macrophages (3.3x); *Tgfb3* was up-regulated in both cell types (2.2x). *Clcf1* was also increased in SCs (2.3x) and macrophages (3.2x). To guide future studies, we determined which, if any, of the cytokines and growth factors increased in 7-month-old mouse neurofibroma SCs also showed increased expression in human plexiform neurofibroma SCs compared to normal human SCs (green boxes in Supplementary Fig. S4, published dataset^40^). *Inhba, Cxcl2, Il1b, Clcf1, Lif*, and *Ccl5* were up-regulated in human neurofibroma SCs, and may justify further study.

Several mouse receptors showed altered expression. As noted above, *Clcf1* expression increased in SCs and macrophages. Expression of the CLCF1 receptor gene *Cntfr* (0.25x) is down-regulated in SCs, suggesting possible compensation. CLCF1 competes with CNTF for binding to and activation of IL6ST (GP130) in complex with CNTFR. *Csf2rb2*, encoding the β subunit common to the IL3, IL5 and CSF2 receptors, was up-regulated both in SCs (11.5x) and macrophages (5.2x). This is of interest given the hyper-reponse of hematopoeietic cells lacking *Nf1* to CSF2. The GDF5/BMP7 receptor gene *Bmpr1b* (2x) and the leptin receptor gene *Lepr* were down-regulated in SCs (2.8x) and macrophages (2.5x).

### Predicted autocrine and paracrine regulations

**Figure 5:**
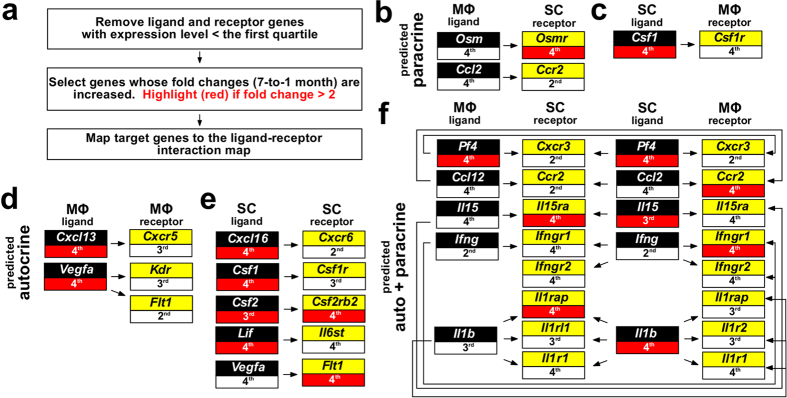
Potential paracrine and autocrine regulations in 7-month-old neurofibroma microenvironment. The relative expression levels are represented as quartiles (1^st^: lowest, 4^th^: highest). DEGs compared to 1-month-old neurofibroma SCs (*Nf1*^*−/−*^) and neurofibroma macrophages (*Nf1*^+*/*+^) are indicated in red boxes (fold >2).

Although the absolute abundance of each mRNA cannot be precisely deduced from gene microarray data only, we used the first quartile (the lowest 25% data points, ~3.1) of overall gene expression intensity as a cutoff to predict absence of gene expression and exclusion from the analysis. Probable autocrine or paracrine regulatory units likely to exist in the 7-month-old neurofibroma microenvironment were then extracted from the ligand-receptor interaction map. This information, together with the increased or decreased expression level of a ligand and/or its corresponding receptor gene in 7-month-old SCs and/or macrophages, predicted several paracrine (Fig. 5a–c) and autocrine (Fig. 5d,e) interactions. Autocrine (SC) and paracrine (SC → macrophage) *Csf1*-*Csf1r* interactions suggest a role for 7-month-old neurofibroma SCs in recruiting/polarizing macrophages within tumor microenvironment. Importantly, autocrine (SC and macrophage) and bi-directional paracrine (SC → macrophage, and macrophage → SC). *Ifng-Ifngr1/2* signals were also predicted (Fig. 5f) and are discussed in more detail below.

**Figure 6:**
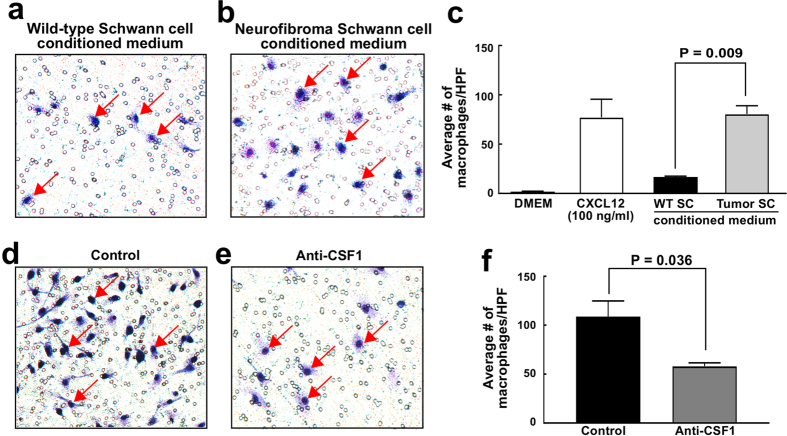
Macrophage migration assay. The number of migrated macrophages (stained in blue) increased significantly in neurofibroma SC conditioned medium compared to the wild-type SC conditioned medium (**a**–**c**). Anti-CSF1 treatment significantly reduced the number of migrated macrophages stimulated by neurofibroma SC conditioned medium (**d**–**f**).

To verify that direct interaction can occur between neurofibroma SCs and macrophages, we performed a macrophage migration assay using SC secreted factors. FACS sorted mouse neurofibroma SCs were briefly cultured, and their conditioned medium was collected. This conditioned medium significantly increased migration of bone marrow derived macrophages (p < 0.009), as compared to wild-type SC conditioned medium, supporting an active role for neurofibroma SC in macrophage accumulation in neurofibromas (Fig. 6a–c). Since CSF1 is a known macrophage chemoattractant and an interaction between CSF1 and is receptor CSF1R (FMS/CD115) was identified in our microarray data analysis (Fig. 5c), we tested if an anti-CSF1 function-blocking antibody might reduce macrophage migration stimulated by neurofibroma SC conditioned medium. Indeed, in 3 experiments a significant decrease was observed (Fig. 6d–f, p < 0.036). Thus, neurofibroma SCs secrete cytokines, including CSF1 that facilitate macrophage migration.

### Transcriptional changes in neurofibroma resemble early stages after sciatic nerve injury

Following crush injury to axons and their associated SCs, P-ERK, a readout of active RAS-GTP signaling, is induced in SCs and persists for 3–5 days^41^. Myelin gene expression, a read-out of SC differentiation, is reduced by day 3 after crush injury. By day 3, macrophages invade the nerve, and proliferation markers such as histone H3 are induced. The expression of *Vegfa* is increased by day 4. Axonal regrowth and re-expression of myelin RNAs begin at day 12.

To test the hypothesis that neurofibroma resembles wounded nerve, we investigated the differential gene expression profiles of mouse and human neurofibroma (compared to normal nerve of each species^42^) to those of 1, 4, 7, and 14 days after rat sciatic nerve injury^43^. DEGs were selected using fold change >3x and FDR q < 0.05 cutoffs from both datasets. Mouse neurofibroma DEGs maximally overlapped with DEGs from day 4 after nerve injury (Supplementary Table S1 and Supplementary Fig. S5), consistent with the hypothesis that neurofibroma resembles early stages after sciatic nerve injury, with injury that fails to resolve. For example, *Ccl5* expression is up-regulated only at day 4 after nerve injury (4.21x), yet expression persists in neurofibroma (4.56x). *Ccl2* expression is up-regulated >80-fold on day 1 after nerve injury, decreasing to 6-fold at days 7 and 14, and remains up-regulated in neurofibroma (2.39x). This finding is consistent with studies of nerves of Raf-ER transgenic mice, in which high levels of P-ERK activation are sustained, and 30.23x elevated levels of *Ccl2* reported^11^.

### Inter- and intra-cellular networks identify inflammation-related regulatory modules

**Figure 7:**
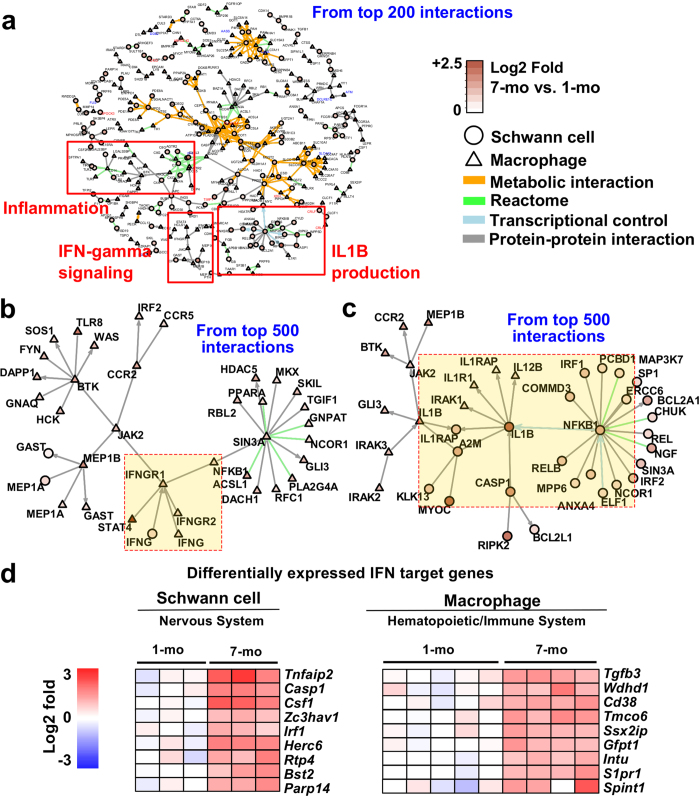
Network analysis and interferon target analysis. (**a**) Intra- and inter-cellular network generated based on top-scoring network interactions revealed sub-networks related to inflammation and immune responses (**b** and **c**). Two functional modules, representing IFN-γ signaling and IL1B production, were re-plotted using a bigger context (top 500 interactions). (**c**) IFN-γ target DEGs from 7-to-1 month comparisons of SC and macrophages were predicted using INTERFEROME v2.0.

The gene/protein network analyses based on the modified NetWalk algorithm^44^ also detected plausible intra- and inter-cellular interactions between 7-month-old neurofibroma SCs and 7-month-old neurofibroma macrophages. Figure 7a displays interactions dominated by metabolic interactions, and immune-related genes and their interactions (red boxes). Three immune- and inflammation-related modules were identified using additional gene set enrichment analysis (Fig. 7a). Two network modules centered on *Ifng* and *Il1b* were re-plotted after extending the networks (Fig. 7b and c).

### Interferons and activated IL1B may promote chronic inflammation in neurofibroma

To test if IFN-γ in neurofibromas might be active, we compared DEGs with identified interferon target genes (http://interferome.org) expressed in peripheral nerve data sets (Fig. 7d). These genes may not be specific for IFN-γ activation; many are targets of both IFN-γ and IFN-α/β in different contexts. Predicted interferon-regulated genes (IRG) were expressed in neurofibroma SCs and macrophages, and differed between the two cell types. Thus IFN-γ may have different downstream effects on gene expression in neurofibroma SCs and neurofibroma macrophages.

**Figure 8:**
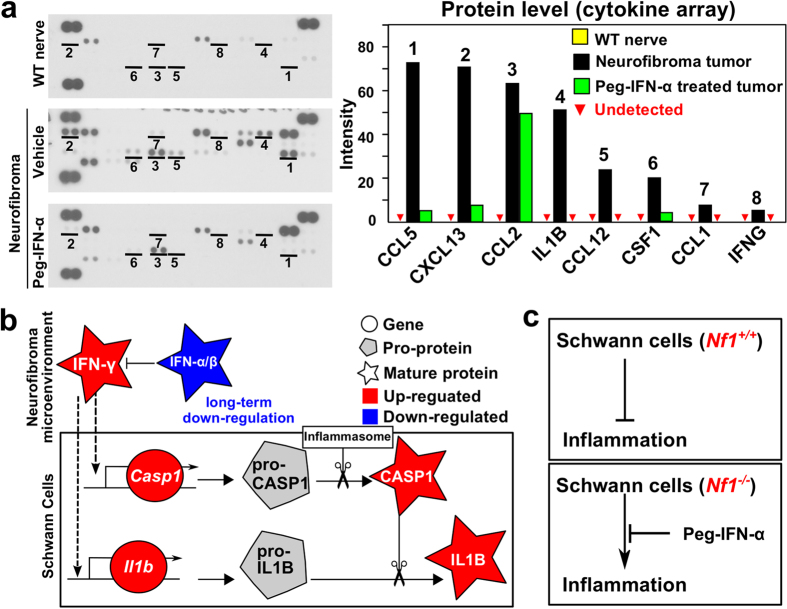
Pro-inflammatory cytokines in Neurofibroma. (**a**) Left panel: Pro-inflammatory cytokines are at low levels in wild-type nerve (top), show increased protein levels in neurofibroma (middle), and are reduced after treatment of neurofibroma with PEGylated IFN-α2b (bottom). Right panel: Relative intensity, reflecting comparative levels of expression for each protein, after the intensity of pixels was averaged and plotted. (**b**) A model developed from gene expression analysis (drawn by Inkscape v0.48, http://inkscape.org). Decreased levels of type-I interferons and increased type-II interferon increase inflammation in the tumor microenvironment by increasing expression of *Casp1* and *Il1b* mRNAs. CASP1 pro-protein is known to be cleaved and thus be activated by the inflammasome. Active CASP1 cleaves pro-IL1B protein, releasing active IL1B cytokine. (**c**) Based on this analysis, normal SCs suppress nerve inflammation. When *Nf1*^*−/−*^ SCs are present, de-regulated interferons result in inflammation, which can be largely normalized by PEGylated IFN-α2b.

Eight pro-inflammatory cytokine mRNAs over-expressed in 7-month-old SCs or macrophages were evaluated for protein expression in mouse neurofibroma tumors, as compared to WT sciatic nerve lysates (Fig. 8a). These included IFN-γ, and its predicted target CSF1. Of note, IL1B and CASP1, the proteinase necessary for cleavage and thus activation of IL1B, were also detected in neurofibroma lysates. To test the idea that imbalance between type-I and type-II inteferons is relevant to inflammation in neurofibromas, we took advantage of the knowledge that IFN-α treatment can reduce IFN-γ levels. We administered PEGylated (stabilized) IFN-α2b to neurofibroma-bearing mice *Nf1*^*fl/fl*^*;DhhCre* mice for 8 weeks (7 to 9 months of age). In this paradigm, MEK inhibition shrinks 75% of neurofibromas, while PEGylated IFN-α2b does not shrink tumors significantly (not shown). IFN-α2b was administered at 10,000 IU weekly, by subcutaneous injection^45^. One day after the last dose, we dissected neurofibromas and measured the relative levels of inflammatory cytokines in neurofibroma lysates. This treatment reduced levels of IFN-γ, IL1B, and CSF1 to, or close to, levels present in wild-type nerve (Fig. 8a). These data suggest that, as predicted by our *in silico* analysis, neurofibroma inflammation can be modulated in an interferon-dependent manner (Fig. 8b,c). Inflammation increases in aged wild-type mice^46^. To exclude the possibility that 7-month-old wild-type mice show increased expression of the inflammatory markers identified in neurofibromas and might account for our findings, we performed qRT-PCR. We chose 5 over-expressed protein genes (*Ccl5, Ccl2, Ccl12, Csf1* and *Il1b*) in Fig. 8a, and monitored their relative mRNA expression in FACS-sorted primary mouse SCs and macrophages in 1-month-old and 7-month-old wild-type mice. Student’s t-tests (p < 0.05) revealed that there was no significant difference in mRNA expression in any of these genes at these time points. *Il1b* was not detectable at either age (Supplementary Fig. S6). Therefore, neurofibroma SCs and macrophages up-regulate inflammatory genes that are not upregulated in wild-type mice at this age.

## Discussion

We describe potential neurofibroma SC-macrophage molecular interactions based on cell type-specific transcriptome analyses. Our findings support the notion that neurofibroma SCs, some of which are *Nf1*^*−/−*^, promote a tumor microenvironment characterized by chronic inflammation, leading to altered gene expression in wild-type stromal cells, including macrophages. Our analysis reveals that neurofibroma SCs and macrophages both progressively adopt pro-inflammatory states during tumor progression, and that nerve and tumor macrophages differ from each other and from previously defined monocyte and macrophage populations. Finally, we find that neurofibroma SCs secrete macrophage chemoattractants including CSF1 and that neurofibromas contain increased levels of numerous additional chemokines, cytokines, and growth factors, including IFN-γ.

We used CD11b^+^ and F4/80^+^ as markers for macrophages in cell sorting, because in tissue sections, 30% of neurofibroma cells express these macrophage markers^14^. We confirmed expression of an additional 12 macrophage marker genes using cluster analysis. It is possible, however, that our macrophage gene expression profiles do not represent all neurofibroma macrophages. For example, rare CD11c^+^ cells are present in neurofibroma and may not be represented^14^. In addition, macrophages are highly plastic cells, and we cannot exclude the possibility that sample processing for FACS altered gene expression patterns. Finally, it remains to be determined if our gene expression profiles are reflective not of a mixed M1/M2 profile but rather of subsets of a larger population. Genes we identified as expressed should enable tests of this hypothesis.

We used p75^+^ cells to sort SCs, and confirmed that the sorted cells express the SC lineage marker *Sox10*, the immature SC/satellite cell marker *Fabp7*, the SC neuregulin receptor *Erbb3*, and the SC myelin markers *Mbp* and *Mag*, among others (Supplementary Fig. S3). We also confirmed that neurofibroma SCs are *Nf1*^*−/−*^ mutant in the *Nf1*^*fl/fl*^*;DhhCre* mouse model, while macrophages are wild-type. P75/Ngfr can label T-cells, but T-cells were excluded from our analysis using light scatter parameters defining cell size along with differences in p75 expression level. Fibroblasts can also express p75 in mouse, but at lower levels than SCs, and the p75^+^ cells we sort are EGFR-negative; fibroblasts express EGFR. We also used p75 to exclude myelinating SCs, which are p75 negative, and thereby obtain p75^+^ Remak bundle SCs and tumor SCs. While cells did express *Mbp* and *Mag*, these may be present at low levels in tumor cells and/or represent contribution of rare myelinating cells. In either case, major contributions of myelinating SCs to the tumor phenotype have been missed in this analysis.

It will be of interest to further characterize and sort tumor SCs and macrophages. We chose two time-points (1 and 7 months) at which to compare gene expression changes because at one month of age, nerve maturation is largely complete. SC myelination is complete, as is Remak bundle formation. Inflammation is known to increase in aged mice (9–15 month old)^46^, and additional age-matched samples may provide additional information. However, we did not detect statistical differences in gene expression between 1- and 7-month-old SCs, or macrophages, in wild-type mouse nerve/DRG (Supplementary Fig. S6a,b) that might account for the increased expression of inflammation-related cytokines and chemokines in neurofibromas.

In addition, it will be important to demonstrate directly that neurofibroma macrophages affect neurofibroma SCs. This may be difficult, given problems in obtaining sufficient neurofibroma macrophages for culture and because macrophages are highly plastic and will alter their phenotypes rapidly upon culture. As a tumor cell’s gene expression profile can be changed dynamically by extracellular signals and stresses, a more detailed time-series analysis should identify changes that occur dynamically in neurofibroma initiation and maintenance, using markers that are validated from the expression analysis. Also, neurofibroma SCs, macrophages, fibroblasts, endothelial cells, and mast cells can contribute to intercellular interactions in the tumor microenvironment, so the cells we sorted are not the only potential sources of signaling molecules in neurofibromas. For example, although type-I interferons are secreted at low levels by most cells, hematopoietic cells, especially plasmacytoid dendritic cells, are a major source of IFN-α, and fibroblasts a major source of IFN-β^47^. It will be worth testing if neurofibroma fibroblasts produce IFN-β, potentially increasing overall levels of type-I interferon in neurofibroma. Furthermore, IFN-γ is generally produced by T-cells, which are rare in neurofibroma; it will be important to test which cells make this factor.

Our gene expression data suggested the possibility that prolonged reduction of IFN-α/β in neurofibroma leads to the expression of IFN-γ and its target genes *Csf1, Lif, Irf1*, and *Casp1* in SCs, possibly contributing to the recruitment and maturation of macrophages. We were able to verify that CSF1 protein is present in neurofibroma lysates, is present in neurofibroma SC medium, and can recruit macrophages. This result is consistent with the finding that blocking the Csf1r decreases macrophage number in the *Nf1^fl/fl^;DhhCre* neurofibroma model^14^ and extends it by showing that at least some neurofibroma CSF1 is made by neurofibroma SCs themselves. We were also able to verify that IFN-γ is increased over wild- type levels in neurofibroma lysates, and Park *et al*.^48^. detected increased levels of IFN-γ in serum from NF1 patients. Low levels of type-I interferon present in neurofibroma might permit pro-inflammatory cytokine protein expression during neurofibroma growth. *Casp1*, a downstream target of IFN-γ^49^ was increased (3.6x); CASP1 protein cleaves pro-IL1B, thereby activating it^50^. IRF1, a key target of interferon, indirectly increases *Il1b* gene expression^51^. SCs differentially express *Irf1* (2.1x), possibly explaining up-regulation of *Il1b* (6.7x) in SCs. This notion is consistent with our finding that PEGylated interferon-alpha-2b (PEG-IFN-α2b) treatment resulted in the decrease of 8 cytokines, including mature IL1B protein, because type-1 interferon can inhibit *Il1b* production^52^. Of note, in a Phase II trial, PEGylated IFN-α2b caused a significant slowdown of neurofibroma growth in some individuals^53^. Our analysis in mice is consistent with and provides a biochemical context for the human studies.

There are similarities between nerve injury, which is followed by recovery of function, and neurofibroma formation. Early after nerve injury SCs express pro-inflammatory cytokines and chemokines, followed by IL1B secretion from SCs. Subsequently, infiltrating macrophages express pro-inflammatory cytokines. Thus, SCs appear to take a leading role in inducing inflammation early after nerve injury, and in neurofibroma. However, we also identify substantial differences between the nerve injury/recovery process and neurofibroma. For example, after peripheral nerve injury Toll-like receptor 2 (TLR2) contributes to chemokine gene expression and macrophage recruitment^54^. TLRs recognize damaged cells and cell debris. In neurofibroma, *Tlr2* is slightly down-regulated (0.78x) in 7-month-old neurofibroma macrophages, and *Ccl2* and *Ccl3*, which can increase *Tlr2* expression, are not significantly up-regulated. Instead, *Tlr8* (5.5x), *Tlr5* (2.7x), and *Tlr9* (~2.0x) are up-regulated; TLR5^55^ and TLR8^56^ relay signals to increase *Il1b* expression. Prolonged exposure to stressors and anti-inflammatory cytokines/chemokines signaling may determine the differential usage of these receptors in neurofibroma.

Another difference between the nerve injury and neurofibroma is the duration of local inflammation. A switch from pro-inflammatory processes such as influx of macrophages to recovery of nerve function is characteristic of nerve injury. In contrast, chronic inflammation without significant apoptosis is characteristic of neurofibroma. The concept that tumors behave as “wounds that do not heal”, stated by H. Dvorak in 1986 57, is reflected in the benign neurofibroma gene signatures we describe. Our findings extend previous understanding, as we show that inflammation increases over time, correlating with nerve tumor formation. Importantly, loss of *Nf1* in SCs does not immediately cause inflammation. Indeed, the interval between loss of the *Nf1* tumor suppressor and tumorigenesis, and increased inflammation, may create a window of opportunity for interfering with tumor formation.

*Nf1*^*−/−*^ SCs must initiate tumorigenesis, as they are the only *Nf1*^*−/−*^ cells present in neurofibromas, but neurofibroma macrophages may maintain the pro-inflammatory state in the neurofibroma microenvironment, accounting for prolonged chronic inflammation. In macrophages, perturbation of the balance between phospho-STAT1 and phospho-STAT3 can redirect signaling. In neurofibroma macrophages, neither *Stat1* nor the *Stat1* target gene *Il10* were differentially expressed; however, phospho-STAT3 is elevated^58^. Given that IFN-γ is elevated in neurofibroma yet IL10 is not, an IFN-γ-dependent STAT1-independent pathway may be relevant^59^. *Stat4* (17x) and *Stat2* (2.7x) were significantly up-regulated and could potentially mediate signaling effects.

Our findings support the idea that SCs and macrophages cross-talk in neurofibroma. The neurofibroma system described here provides a platform upon which to investigate temporal and mechanistic aspects of RAS/interferon signaling. Finally, our study provides a wealth of data for future investigation of individual genes and processes in neurofibroma.

## Methods

### Ethics statement

All experiments with vertebrate animals were performed in accordance with Institutional guidelines and regulations at the Cincinnati Children’s Hospital Medical Center (CCHMC), and methods were approved by the CCHMC Institutional Review Board.

### Mice

All mice were maintained on the C57Bl/6 background from Harlan laboratories (Indianapolis, IN), by in-house breeding to *Nf1*^*fl/*+^ and *DhhCre* to obtain *Nf1*^*fl/fl*^*;DhhCre* or *Nf1*^*fl/fl*^ mice, as previously described^3^. Mouse genotyping and recombination assays were carried out as described^2^.

### Cell dissociation for cell sorting

We collected mouse DRG/neurofibroma/nerve, cut tissue into 1–3 mm^3^ pieces, and plated them in dissociation medium containing 20 mL L-15 (Mediatech), 0.5 mg/mL collagenase type 1 (Worthington; Lakewood, NJ), and 2.5 mg/mL dispase protease type II (Cambrex; East Rutherford, NJ) at 37 °C for 4–6 hours with shaking as described^58^. The dissociation reaction was stopped by adding Dulbecco’s Modified Eagle Medium (DMEM) + 10% fetal bovine serum (FBS). Undigested DRG and tumors were excluded using a 100 μM cell strainer. Cells were collected by centrifugation.

### Cell sorting

We incubated the dissociated mouse DRG/neurofibroma cell suspensions with anti-mouse monoclonal antibodies against CD11b (8G12/HPCA-2, Becton–Dickinson; San Jose, CA) bound to allophycocyanin (APC) anti-p75/NGFR (C40-1457, Becton–Dickinson) bound to phycoerythrin (PE), anti-F4/80 bound to Cy5.5 on ice in a solution containing phosphate-buffered saline (PBS)/0.2%BSA/0.01% NaN_3_ for 30 minutes. After washing, we resuspended cells in PBS/0.2%BSA/0.01% NaN_3_/2 mg/mL 7-aminoactinomycin D (7-AAD, Invitrogen). We carried out isotopic controls with irrelevant IgG1–APC, IgG1–PE and IgG1-Cy5.5 in parallel. We acquired cell suspensions in a dual-laser (Argon 488 and dye laser 630 or HeNe 633) FACSCanto (Becton–Dickinson) and analyzed on an “alive” gate based on light scatter parameters and 7-AAD staining negativity. Because some T cells are p75 positive, our forward scaffold enable us to avoid T cells when sorting SCs.

### RNA purification

RNAs were isolated using RNeasy mini kit (QIAGEN, Valencia, CA). RNA purification was performed as described. RNA integrity was determined by Agilent BioAnalyzer. RNAs with RNA Integrity Number (RIN) ≥ 9 were processed for Affymetrix platform.

### Microarrays

For each microarray (SCs, macrophages), Affymetrix GeneChip Command Console (v4.0.0) was used to create .chp files. All the probe sets on Affymetrix Mouse Gene 2.0 ST array (Mogene-2_0-st-v1.na33.2.mm10) were summarized by the Affymetrix Expression Console program (v1.3.1) using robust multi-chip average (RMA) method. After preprocessing steps, data from two batches were combined and their batch effects were corrected using ComBat method implemented in Bioconductor’s sva package. HUGO Gene Nomenclature Committee (HGNC)’s orthology prediction database (http://www.genenames.org/cgi-bin/hcop) was used to get human-to-mouse gene orthology information. Mouse genes with strong human orthologs were included in this study. Microarray raw data are available (Accession Number: GSE78901) at Gene Expression Omnibus (GEO).

### Differential gene expression

The Bioconductor/R limma package was used to define DEGs between two groups. Genes were considered differentially expressed when they passed two cutoff criteria (FDR q < 0.05 and fold change >2x). The most updated MGI (for mouse, http://www.informatics.jax.org) and HGNC (for human, http://www.genenames.org) gene/protein nomenclature was adopted in this study.

### Gene set enrichment analysis

Gene set enrichment analysis was performed using the WebGestalt webserver (http://bioinfo.vanderbilt.edu/webgestalt/). DEG sets were queried against the KEGG database and and FDR q < 0.05 cutoff was applied to select significantly enriched KEGG pathways.

### Ligand-Receptor interaction map

To construct a ligand-receptor interaction map, we compiled three separate public databases providing ligand-receptor binding-pair annotations. To collect a list of ligand and receptor genes, we parsed Gene Ontology (GO) terms associated with extracellular ligands and membrane receptors. The Database of Ligand-Receptor Partners (DLRP, http://dip.doe-mbi.ucla.edu/dip/DLRP.cgi) includes 462 interactions between 176 ligands and 133 receptors. Experimentally proven interactions (*in vivo* and/or *in vitro*) extracted from BioGrid v3.2 (http://thebiogrid.org) include 64 interactions between 36 ligands and 107 receptors. An XML file containing 242 cytokine-cytokine receptor interactions (138 ligands and 107 receptors) was downloaded from KEGG (mmu:04062) and parsed. After deleting redundant interaction pairs, we compiled an interaction map containing 635 ligand-receptor interactions including 182 ligands and 205 receptor genes. DEGs from the comparison of 7-month-old SC (*NF1*^*−/−*^) group to 1-month-old SC and 7-month-old macrophages group to 1-month-old DRG macrophages by applying FDR q < 0.05 and fold change >2x cutoffs, and then mapped to this ligand-receptor map. The final interaction map was automatically generated using in-house Perl script and the GraphViz graph package (http://www.graphviz.org).

### Macrophage subtype gene expression data

Gene Expression datasets of macrophage/monocyte subtypes (n = 23) were downloaded from the Immunological Genome Project (ImmGen) data portal (https://www.immgen.org/). This includes bone marrow classical monocytes, bone marrow non-classical monocytes, bone marrow macrophages, red pulp macrophages, lung residential macrophages, peritoneal dendritic cells, and small intestine dendritic cells. To characterize the subtype(s) of our 1- and 7-month-old neurofibroma macrophages, we applied Exploratory Factor Analysis (EFA)^23^ to our data and to the ImmGen datasets, using total transcriptomes, ligand-receptor genes from our re-compilation, and M1/M2 polarization signature genes. M1/M2 polarization signature gene sets were collected from published papers^19^,^20^,^21^,^22^. The number of factors was determined by Velicer’s minimum average partial (MAP) procedure in R (psych package), and maximum-likelihood factor analysis was performed using factanal function (stats package) in R.

### TAM gene expression data

We compared monocyte/macrophage datasets to those available in the ImmGen project (GSE37448) and TAM datasets, including glioma, neuroblastoma, and thymoma (GSE59047) to 1- and 7-month-old neurofibroma macrophages. To identify hidden clusters, exploratory factor analysis (EFA)^23^ was applied using gene expression profiles from total transcriptomes, ligand-receptor genes from our re-compilation, and M1/M2/TAM polarization signature genes^19^.

### Macrophage migration assay

We used 24-well Transwells (Corning #3421, New York, NY, 5.0 μm pore size) for migration assays. We added 0.6 mL mouse wild-type SC or neurofibroma SC conditioned medium, collected from 3–5 × 10^6^ cells cultured in DMEM + 10% FBS + Forskolin + β-heregulin (HRG) for 18 hours at 80–90% confluence to wells, then added a Transwell insert, and 0.1 mL DMEM medium containing 10% FBS or 50 ng/mL Anti-CSF1 (R&D Systems MAB4161) in the same medium. Then, 1.2 × 10^4^ bone marrow-derived macrophages were added to the inside compartment of the Transwell insert. DMEM with and without 100 ng CXCL12/SDF-1α (460-SD-010: R&D Systems) were used as negative and positive controls, respectively. After 24 hours at 37 °C, 5% CO_2_, non-migrated cells were removed from the upper surface of the membrane by scrubbing using cotton tipped swabs. Cells on the lower surface of the membrane (migrated) were fixed in 100% methanol for 2 minutes, then stained with Giemsa for 1 hour. The Transwell inserts were washed in distilled water twice, air-dried overnight, and viewed and imaged under a Leica dissection microscope.

### Gene network analysis using NetWalk

A modified version of NetWalk algorithm^44^ was used to handle bi-cellular interaction. Briefly, we calculated t-values using limma by comparing 7-month-old *Nf1*^*−/−*^ SCs to 1-month-old *Nf1*^*−/−*^ SCs and 7-month-old neurofibroma macrophages to 1-month-old DRG macrophages. The resulting t-values were transformed by quantile normalization to obtain identical distributions for two sets and used as node weights for the NetWalk analyses.

### PEGylated interferon alpha-2b treatment

PEGylated interferon alpha-2b was purchased at the Cincinnati Children’s Hospital (National Drug Code 00085132302) pharmacy. Each RediPen was brought to room temperature for at least 30 minutes. Contents (50 mcg per 0.5 ml) were diluted with sterile 1x phosphate buffered saline (pH 7.4) so that each 25 gram mouse received 10,000 I.U. in 100 μl 1X/week by subcutaneous injection, or a similar volume of vehicle^45^. One RediPen was used for injections on a single day. Volume was adjusted according to weight. Treatment was continued for 8 doses. We examined tumor bearing animals of both sexes for therapeutic response to PEGylated interferon alpha-2b (n = 19) as described, using volumetric magnetic resonance imaging (MRI) to evaluate tumor growth^60^. Mice were monitored daily and weighed weekly; no mice lost >10% body weight or required sacrifice. At the end of the treatment trial, we removed neurofibromas and froze them at −80 °C.

### Mouse cytokine array analysis

Mouse cytokine protein expressions were quantified using mouse cytokine array (panel A, R&D system, Minneapolis, MN). Briefly, proteins were extracted from *Nf1*^*fl/fl*^*;DhhCre* mouse neurofibromas and *Nf1*^*fl/fl*^ mouse sciatic nerves. Arrays were performed according to the instructions provided by R&D System on 200 μg lysate protein. The intensities of the white dots that were converted from the original black dots were measured using ImageJ software.

## Additional Information

**How to cite this article**: Choi, K. *et al*. An inflammatory gene signature distinguishes neurofibroma Schwann cells and macrophages from cells in the normal peripheral nervous system. *Sci. Rep.* **7**, 43315; doi: 10.1038/srep43315 (2017).

**Publisher's note:** Springer Nature remains neutral with regard to jurisdictional claims in published maps and institutional affiliations.

## Supplementary Material

Supplementary Figures and Tables
