# Supplementary material for: An inflammatory gene signature distinguishes neurofibroma Schwann cells and macrophages from cells in the normal peripheral nervous system
Source: Sci Rep. 2017 Mar 3;7:43315. doi: 10.1038/srep43315 (PMC5335359; doi:10.1038/srep43315)
Supplement: Supplementary Figures and Tables [file srep43315-s1.pdf]

Supplementary Figures and Tables

An inflammatory gene signature distinguishes neurofibroma Schwann cells and macrophages from cells in the normal peripheral nervous system

Kwangmin Choi[1], Kakajan Komurov[1], Jonathan S. Fletcher[1], Edwin Jousma[1], Jose A. Cancelas[1,2], Jianqiang Wu[1*], and Nancy Ratner[1*]

[1]Division of Experimental Hematology and Cancer Biology, Cancer and Blood Diseases Institute, Cincinnati Children's Hospital Medical Center, Dept. of Pediatrics, University of Cincinnati, Cincinnati, OH 45229, USA

[2]Hoxworth Blood Center, College of Medicine, University of Cincinnati, Cincinnati, OH 45229, USA

[*]**Correspondence to:** Nancy.Ratner@cchmc.org or Jianqiang.Wu@cchmc.org

Phone: 513-636-9469 (Nancy Ratner), 513-636-0955 (Jianqiang Wu)

Fax: 513-636-3549

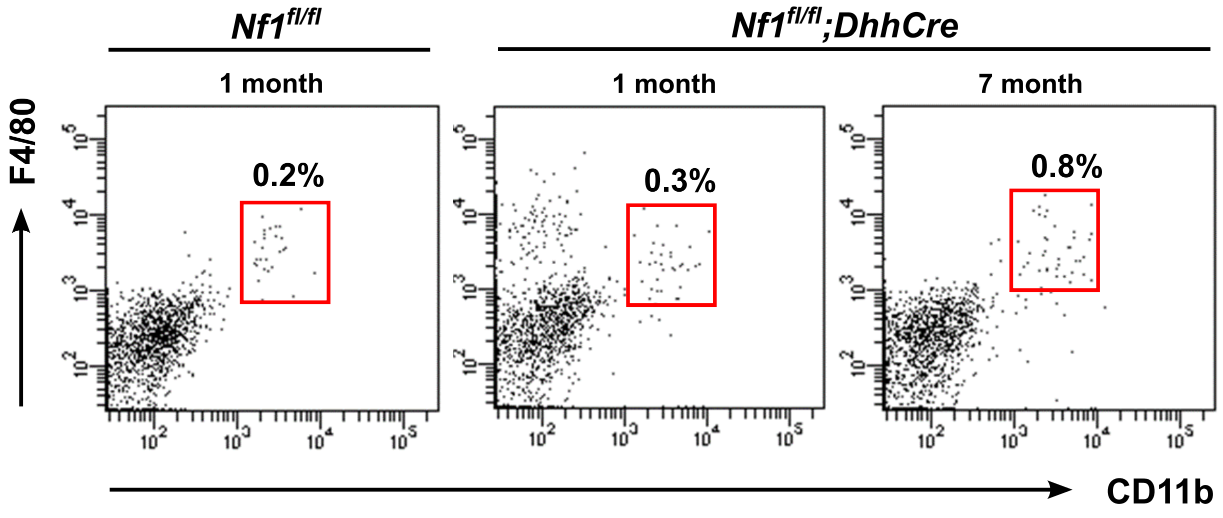

Supplementary Figure S1: **Macrophage content is increased in 7-month-old mouse neurofibromas.** Representative example of flow-cytometry analysis shows populations of F4/80$^+$;CD11b$^+$ macrophages (red square) isolated from 1-month-old *Nf1*$^{fl/fl}$ (left), 1-month-old *Nf1*$^{fl//fl}$*;DhhCre* (middle), and 7-month-old *Nf1*$^{fl//fl}$*;DhhCre* mouse DRG/nerve, with neurofibroma if present, after *in vitro* dissociation. Some mice also contained F4/80$^-$;CD11b$^+$ macrophages, but this varied from mouse to mouse.

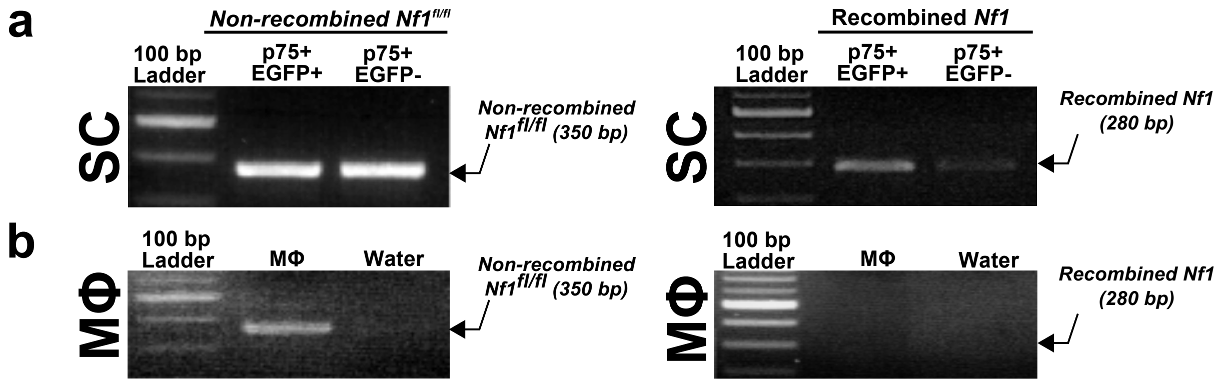

Supplementary Figure S2: ***Nf1* Cre-mediated recombination occurs in Schwann cells, not macrophages.** FACS sorted p75^{+}EGFP^{+} and p75^{+}EGFP^{-} SC populations are *Nf1*$^{fl/fl}$. p75^{+}EGFP^{+} SCs show *Nf1* recombination; p75^{+}EGFP^{-} SCs showed less recombination. Macrophages do not show *Nf1* recombination. The same amounts of DNA amplified by PCR. Genotyping was performed as described in: Zhu Y et al. Genes Dev. 2001;15(7):859-76.

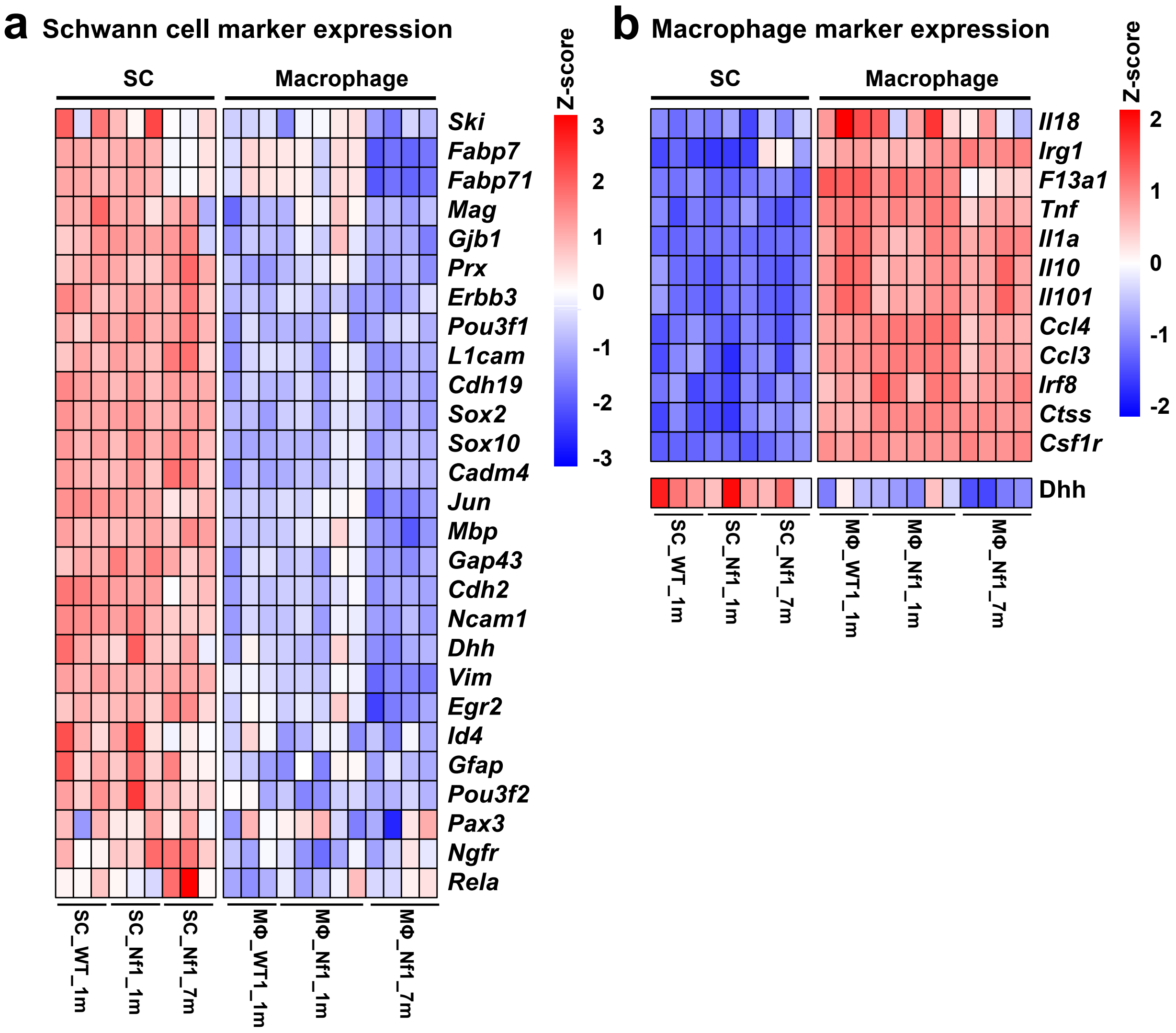

Supplementary Figure S3: **Sorted Schwann cells (a) and macrophages (b) express characteristic RNAs.** Schwann cell markers (**a**) were collected from Mirsky R, *et al*., J Peripher Nerv Syst. 2008; 13(2):122-35), and include the SC marker *Dhh*.

Macrophage markers (**b**) were collected from R&D systems' public resource (https://www.rndsystems.com/research-area/macrophage-markers). Colors in these heatmaps represent Z-score: z = (X - μ) / σ, where z is the z-score, X is the value of the element, μ is the population mean, and σ is the standard deviation.

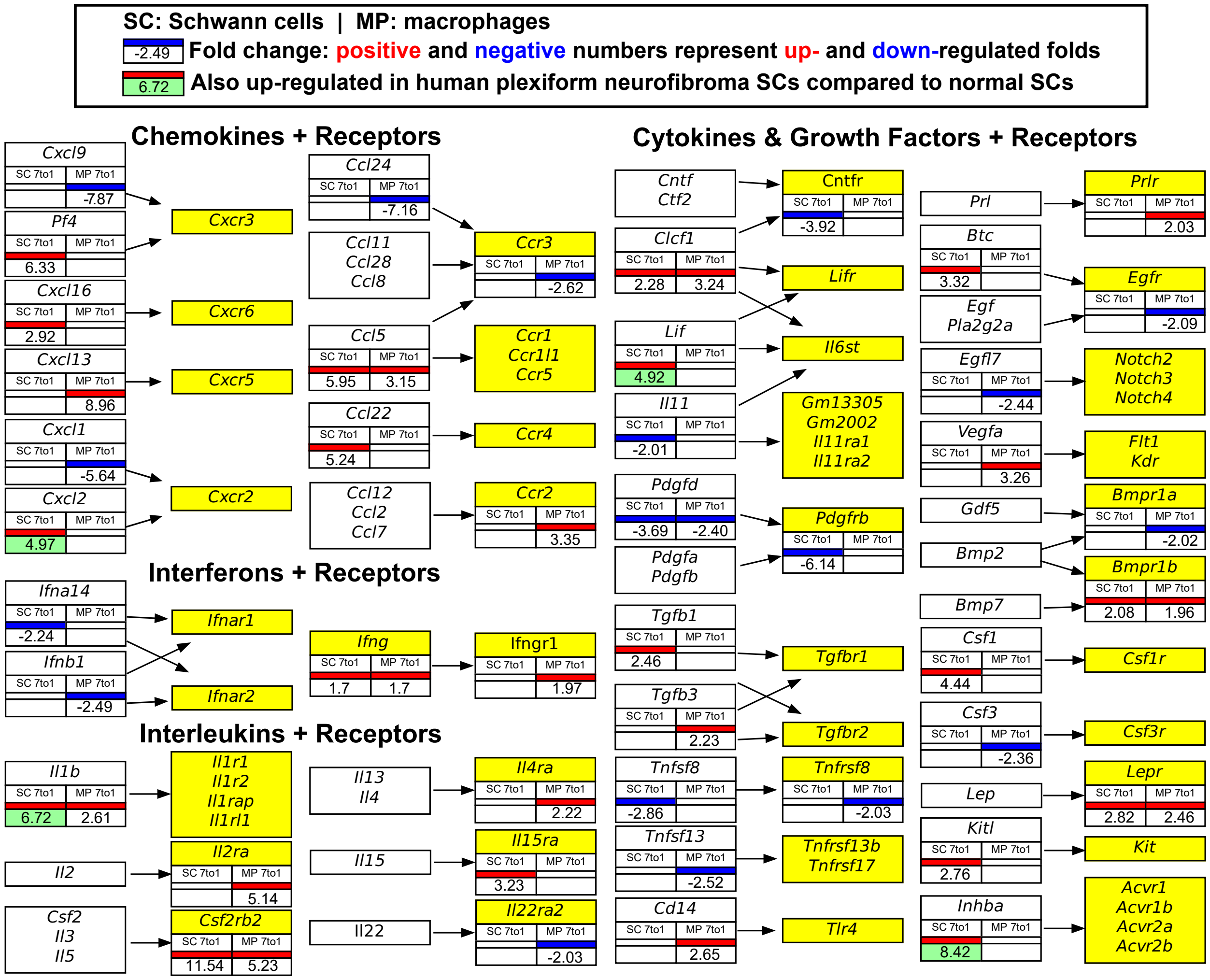

Supplementary Figure S4: Ligand-receptor interaction interface map Ligand (white boxes)-receptor (yellow boxes) pairs are shown if at least one gene in a given ligand-receptor pair was differentially expressed (fold change > ~2x and FDR <0.05). Numbers in each gene box represent fold change, with up- (red) or down-regulated (blue) fold changes shown in base 10.

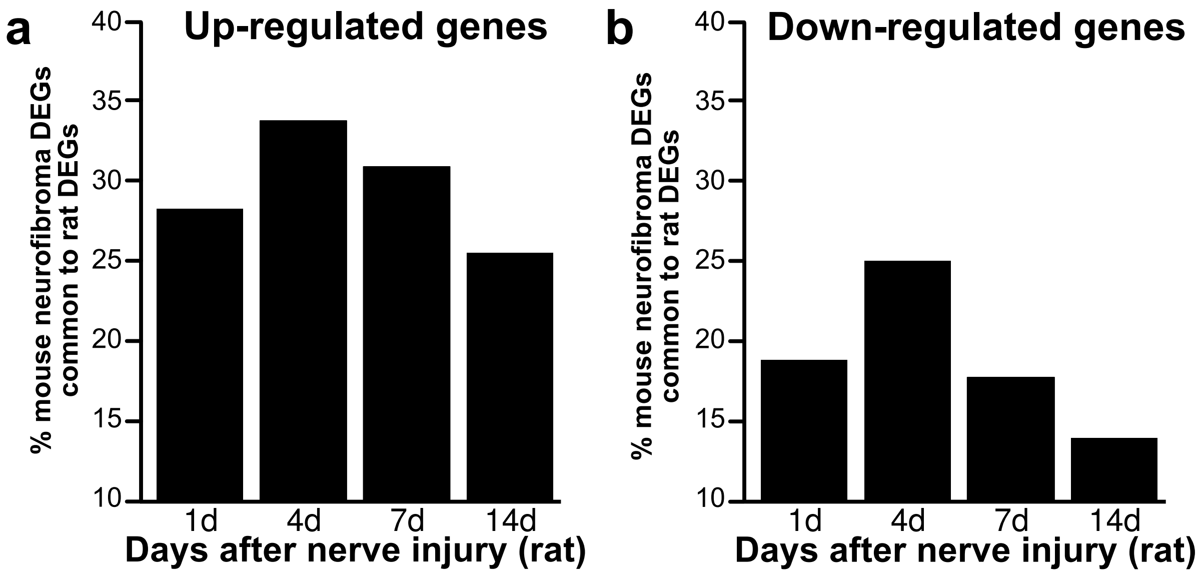

Supplementary Figure S5: **The percentage of mouse neurofibroma DEGs common to DEGs from crushed rat sciatic nerve (compared to uninjured nerve).** Up-regulated rat DEGs at Day 1, 4, 7, and 14 after rat nerve injury overlapped with 28, 33, 31, and 26% of the 170 up-regulated mouse neurofibroma DEGs, respectively. Down-regulated rat DEGs at Day 1, 4, 7, and 14 after rat nerve injury were overlapped with 19, 25, 18, and 14% of the 368 down-regulated mouse neurofibroma DEGs, respectively.

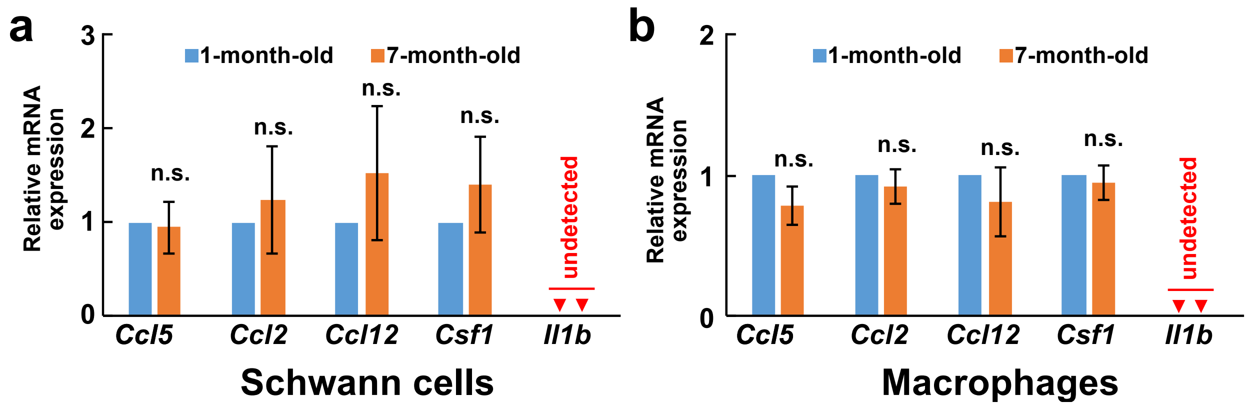

Supplementary Figure S6: **Expression of Inflammatory Cytokine mRNAs do not change in wild-type nerve/DRG between 1 and 7 months of age.** We used FACS sorted wild-type mouse Schwann cells **(a)** and wild-type mouse macrophages **(b)** to generate mRNA**.** We used qRT-PCR to evaluate the relative expression levels of *Ccl5*, *Ccl2*, *Ccl12*, *Csf1* and *Il1b* in 1-month-old (blue, n=3 groups, 5 mice/group) and 7-month-old (orange, n=3 experiments, 5 mice/group) wild-type mice. The relative mRNA expression was normalized to the average of 1-month-old mice. Data is shown as the average +/- S.E.M. of 3 experiments. *Il1b* mRNA is not detectable either in SCs or macrophages in wild-type mice (n.s. = no significant difference = Student's t-test p > 0.05)

Supplementary Table S1: Differential gene expression patterns in mouse neurofibroma versus rat sciatic nerve during injury and recovery

# Up-regulated in Both (> 3 fold, FDR q < 0.05)

Neurofibroma vs nerve		Day1 after nerve injury	
**Mouse**	**FC**	**Rat**	**FC**
Aif1	4.46	Aif1	12.13
Ajap1	3.04	Ajap1	6.19
Apobec1	3.37	Apobec1	16.59
Btc	5.86	Btc	11.24
Ccl12	3.81	Ccl12	28518.46
Ccnb1	3.65	Ccnb1	7.71
Cd44	4.15	Cd44	6.99
Cdkn3	3.44	Cdkn3	4.56
Clec5a	3.49	Clec5a	11.11
Coro1a	3.53	Coro1a	14.7
Cxcl10	4.58	Cxcl10	8.63
Cxcl14	3.29	Cxcl14	18.22
Cytip	3.33	Cytip	11.71
Ereg	24.32	Ereg	120.37
F2rl1	6.25	F2rl1	6.11
Fgr	3.82	Fgr	22.27
Fyb	3.41	Fyb	7.4
Glipr1	3.11	Glipr1	13.96
Gp1bb	3.09	Gp1bb	127.21
Gpnmb	3.97	Gpnmb	32.32
Gpr35	6.99	Gpr35	6.19
Gpr65	3.31	Gpr65	14.48
Havcr2	4.14	Havcr2	16.09
Hck	4.73	Hck	13.35
Hcls1	3.19	Hcls1	10.17
Ifit1	4.41	Ifit1	59.52

Kcnn4	7.12		Kcnn4	18.17
Klk8	11.23		Klk8	123.37
Klrd1	4.46		Klrd1	6.99
Ly86	6.14		Ly86	6.92
Mcm6	3.22		Mcm6	8.5
Myh1	5.07		Myh1	134.35
Oasl2	4.28		Oasl2	11.97
Pkib	6.2		Pkib	4.66
Pou2af1	3.81		Pou2af1	3.41
Ptpn22	3.12		Ptpn22	3.49
Pttg1	3.32		Pttg1	4
Rps6ka6	3.25		Rps6ka6	3.49
Sash3	3.21		Sash3	8.69
Selplg	3.43		Selplg	9.23
Siglec5	7.02		Siglec5	8.71
Slamf8	3.51		Slamf8	10.36
Slc14a1	5.65		Slc14a1	12.23
Slc15a3	3.6		Slc15a3	6.96
Steap1	3.21		Steap1	104.18
Tagln2	4.4		Tagln2	3.58
Timp1	7.18		Timp1	8.5
Tnc	4.79		Tnc	5.49
Vav1	3.02		Vav1	13.89

Down-regulated in Both (> 3 fold, FDR q < 0.05)

**Neurofibroma vs nerve**			**Day1 after nerve injury**	
**Mouse**	**FC**		**Rat**	**FC**
Acvr1c	0.13		Acvr1c	0.05
Adcy1	0.14		Adcy1	0.29
Adh1	0.32		Adh1	0.02
Adipoq	0.05		Adipoq	0.14
Akr1c14	0.28		Akr1c14	0.24
Alb	0.27		Alb	0.25
Aoc3	0.13		Aoc3	0.25
Aqp7	0.22		Aqp7	0.11
Aspn	0.29		Aspn	0.21
C1qtnf9	0.27		C1qtnf9	0.19

Car3	0.05
Ccdc80	0.31
Cdo1	0.17
Cidec	0.07
Cldn19	0.26
Cldn22	0.17
Col23a1	0.22
Crybb1	0.25
Cyb5r2	0.25
Cyp2e1	0.05
Ddn	0.26
Dmrt2	0.23
Dpep1	0.25
Dpt	0.09
Drp2	0.26
Efnb3	0.23
Egfl6	0.27
Eln	0.33
Emid1	0.28
Extl1	0.21
Fa2h	0.19
Fgf4	0.09
Fmod	0.16
Gdf10	0.17
Gsta3	0.2
Kcnk1	0.26
Lect1	0.29
Lep	0.04
Lgals12	0.27
Limch1	0.3
Mag	0.26
Mal	0.3
Mc2r	0.2
Mettl7b	0.28
Mrap	0.29
Myrip	0.3
Nnat	0.27
Ogn	0.29
Pck1	0.15

Car3	0.17
Ccdc80	0.23
Cdo1	0.1
Cidec	0.05
Cldn19	0.27
Cldn22	0.18
Col23a1	0.18
Crybb1	0.16
Cyb5r2	0.2
Cyp2e1	0.14
Ddn	0.1
Dmrt2	0.24
Dpep1	0.08
Dpt	0.31
Drp2	0.32
Efnb3	0.11
Egfl6	0.07
Eln	0.11
Emid1	0.25
Extl1	0.31
Fa2h	0.15
Fgf4	0.15
Fmod	0.1
Gdf10	0.27
Gsta3	0.13
Kcnk1	0.23
Lect1	0.28
Lep	0.04
Lgals12	0.04
Limch1	0.32
Mag	0.27
Mal	0.23
Mc2r	0.09
Mettl7b	0.1
Mrap	0.23
Myrip	0.17
Nnat	0.25
Ogn	0.18
Pck1	0.03

Pla2g5	0.08		Pla2g5	0.11
Pllp	0.24		Pllp	0.25
Prx	0.23		Prx	0.28
Ptger3	0.26		Ptger3	0.32
Rbp7	0.27		Rbp7	0.14
Reep6	0.31		Reep6	0.18
Retn	0.08		Retn	0.15
Rhobtb3	0.23		Rhobtb3	0.24
Sema4g	0.17		Sema4g	0.18
Sema5a	0.18		Sema5a	0.21
Slc15a2	0.24		Slc15a2	0.13
Slc36a2	0.17		Slc36a2	0.21
Slc6a1	0.13		Slc6a1	0.1
Slc7a10	0.31		Slc7a10	0.06
Smtn	0.25		Smtn	0.22
Spon2	0.28		Spon2	0.3
St8sia5	0.15		St8sia5	0.15
Sult1a1	0.32		Sult1a1	0.07
Thrsp	0.3		Thrsp	0.03
Timp4	0.16		Timp4	0.17
Tmem40	0.21		Tmem40	0

Up-regulated in Both (> 3 Fold, FDR q < 0.05)

Neurofibroma vs nerve			Day4 after nerve injury	
**Mouse**	**FC**		**Rat**	**FC**
Aif1	4.46		Aif1	10.05
Ajap1	3.04		Ajap1	13.62
Apobec1	3.37		Apobec1	20.25
Bdnf	5.73		Bdnf	3.38
Btc	5.86		Btc	20.45
Ccl12	3.81		Ccl12	5438.79
Ccl5	4.56		Ccl5	4.21
Ccnb1	3.65		Ccnb1	12.41
Ccnd1	3.86		Ccnd1	3.74
Cd3e	3.56		Cd3e	14.62
Cd44	4.15		Cd44	5.85
Cdkn2a	10.21		Cdkn2a	3.96

Cdkn3	3.44
Clec5a	3.49
Coro1a	3.53
Crmp1	6.51
Cxcl10	4.58
Cxcl14	3.29
Cytip	3.33
F2r	3.59
F2rl1	6.25
Fgr	3.82
Fyb	3.41
Glipr1	3.11
Gp1bb	3.09
Gpnmb	3.97
Gpr35	6.99
Gpr65	3.31
Havcr2	4.14
Hck	4.73
Hcls1	3.19
Hs6st2	4.29
Itga10	3.01
Kcnn4	7.12
Klrd1	4.46
Ltbp2	3.15
Ly86	6.14
Mcm6	3.22
Ms4a4c	5.59
Myh1	5.07
Neto2	3.29
Oasl2	4.28
Pkib	6.2
Ptpn22	3.12
Pttg1	3.32
Sash3	3.21
Selplg	3.43
Siglec5	7.02
Slamf8	3.51
Slc14a1	5.65
Slc15a3	3.6

Cdkn3	6.3
Clec5a	6.6
Coro1a	8.56
Crmp1	3.81
Cxcl10	8.4
Cxcl14	4.56
Cytip	11.21
F2r	3.09
F2rl1	3.86
Fgr	9.6
Fyb	4.47
Glipr1	6.94
Gp1bb	95.72
Gpnmb	104.3
Gpr35	5.13
Gpr65	12.36
Havcr2	12.52
Hck	12.9
Hcls1	8.01
Hs6st2	3.01
Itga10	4.34
Kcnn4	23.78
Klrd1	16.04
Ltbp2	5.17
Ly86	5.72
Mcm6	7.63
Ms4a4c	12.92
Myh1	8.57
Neto2	4.01
Oasl2	5.54
Pkib	4.12
Ptpn22	5.43
Pttg1	5.4
Sash3	7.39
Selplg	4.93
Siglec5	4.36
Slamf8	6.86
Slc14a1	9.46
Slc15a3	8.07

Sox11	7.99		Sox11	3.86
Steap1	3.21		Steap1	47.27
Timp1	7.18		Timp1	3.17
Tnc	4.79		Tnc	16.94
Vav1	3.02		Vav1	17.58

Down-regulated in Both (> 3 fold, FDR q < 0.05)

Neurofibroma vs nerve			Day4 after nerve injury	
**Mouse**	**FC**		**Rat**	**FC**
Acvr1c	0.13		Acvr1c	0.04
Adcy1	0.14		Adcy1	0.32
Adh1	0.32		Adh1	0.01
Adipoq	0.05		Adipoq	0.05
Alb	0.27		Alb	0.08
Aoc3	0.13		Aoc3	0.32
Aqp7	0.22		Aqp7	0.07
C1qtnf9	0.27		C1qtnf9	0.32
Car3	0.05		Car3	0.06
Car4	0.14		Car4	0.16
Casq2	0.31		Casq2	0.25
Cdo1	0.17		Cdo1	0.08
Cidec	0.07		Cidec	0.02
Clcn2	0.25		Clcn2	0.15
Cldn19	0.26		Cldn19	0.08
Cldn22	0.17		Cldn22	0.25
Cntf	0.3		Cntf	0.15
Col23a1	0.22		Col23a1	0.05
Cryaa	0.23		Cryaa	0.04
Crybb1	0.25		Crybb1	0.06
Ctnnal1	0.23		Ctnnal1	0.29
Cttnbp2	0.29		Cttnbp2	0.21
Cyb5r2	0.25		Cyb5r2	0.07
Cyp2e1	0.05		Cyp2e1	0.12
Ddah1	0.22		Ddah1	0.09
Ddn	0.26		Ddn	0.04
Dmrt2	0.23		Dmrt2	0.09
Dpep1	0.25		Dpep1	0.13

Drp2	0.26
Efnb3	0.23
Egfl6	0.27
Eln	0.33
Emid1	0.28
Extl1	0.21
Fa2h	0.19
Fgf4	0.09
Fgf7	0.24
Filip1	0.17
Fmod	0.16
Gdf10	0.17
Ghr	0.25
Gjb1	0.15
Gpm6a	0.31
Jakmip2	0.33
Kcnk1	0.26
Kif13b	0.24
Krt19	0.2
Lect1	0.29
Lep	0.04
Lgals12	0.27
Limch1	0.3
Lipe	0.28
Lpl	0.15
Mag	0.26
Mal	0.3
Mc2r	0.2
Mettl7b	0.28
Mgll	0.23
Mme	0.16
Mrap	0.29
Myrip	0.3
Nrgn	0.28
Ogn	0.29
Pck1	0.15
Pla2g5	0.08
Pllp	0.24
Prr15	0.23

Drp2	0.13
Efnb3	0.15
Egfl6	0.08
Eln	0.08
Emid1	0.09
Extl1	0.24
Fa2h	0.04
Fgf4	0.02
Fgf7	0.26
Filip1	0.25
Fmod	0.09
Gdf10	0.16
Ghr	0.25
Gjb1	0.1
Gpm6a	0.18
Jakmip2	0.22
Kcnk1	0.05
Kif13b	0.29
Krt19	0.19
Lect1	0.05
Lep	0.02
Lgals12	0.04
Limch1	0.11
Lipe	0.28
Lpl	0.24
Mag	0.07
Mal	0.09
Mc2r	0.08
Mettl7b	0.08
Mgll	0.2
Mme	0.26
Mrap	0.06
Myrip	0.12
Nrgn	0.21
Ogn	0.31
Pck1	0.03
Pla2g5	0.08
Pllp	0.14
Prr15	0.21

Prss12	0.26
Prx	0.23
Rapgef3	0.32
Rbp4	0.07
Rbp7	0.27
Reep6	0.31
Retn	0.08
Rhobtb3	0.23
Sema4g	0.17
Sema5a	0.18
Slc15a2	0.24
Slc36a2	0.17
Slc6a1	0.13
Slc6a4	0.31
Slc7a10	0.31
Smtn	0.25
St8sia5	0.15
Sucnr1	0.25
Sult1a1	0.32
Tesk2	0.28
Thrsp	0.3
Timp4	0.16
Tmem27	0.17
Tmem40	0.21

Prss12	0.2
Prx	0.08
Rapgef3	0.29
Rbp4	0.22
Rbp7	0.2
Reep6	0.07
Retn	0.07
Rhobtb3	0.16
Sema4g	0.08
Sema5a	0.07
Slc15a2	0.12
Slc36a2	0.07
Slc6a1	0.19
Slc6a4	0.15
Slc7a10	0.18
Smtn	0.27
St8sia5	0.07
Sucnr1	0.26
Sult1a1	0.07
Tesk2	0.33
Thrsp	0.01
Timp4	0.14
Tmem27	0.01
Tmem40	0.08

Up-regulated in Both (> 3 fold, FDR q < 0.05)

Neurofibroma vs nerve	
**Mouse**	**FC**
Aif1	4.46
Ajap1	3.04
Apobec1	3.37
Btc	5.86
Ccl12	3.81
Ccl5	4.56
Ccnb1	3.65
Cd3e	3.56
Cd44	4.15

Day7 after nerve injury	
**Rat**	**FC**
Aif1	5.29
Ajap1	11.29
Apobec1	8.48
Btc	15.06
Ccl12	3169.42
Ccl5	3.38
Ccnb1	4.66
Cd3e	6.62
Cd44	4.07

Cdkn2a	10.21
Clec5a	3.49
Coro1a	3.53
Cst6	3.46
Cxcl10	4.58
Cxcl14	3.29
Cytip	3.33
F2rl1	6.25
Fgr	3.82
Fyb	3.41
Gabrb2	4.54
Gfra3	9.8
Glipr1	3.11
Gpnmb	3.97
Gpr35	6.99
Gpr65	3.31
Havcr2	4.14
Hck	4.73
Hcls1	3.19
Ifit1	4.41
Itga10	3.01
Kcnj10	14.34
Kcnn4	7.12
Klrd1	4.46
Ltbp2	3.15
Ly86	6.14
Mcm6	3.22
Ms4a4c	5.59
Myh1	5.07
Neto2	3.29
Oasl2	4.28
Pkib	6.2
Ptpn22	3.12
Ptprz1	13.22
Sash3	3.21
Selplg	3.43
Siglec5	7.02
Slamf8	3.51
Slc14a1	5.65

Cdkn2a	3.52
Clec5a	4.13
Coro1a	4.86
Cst6	6.43
Cxcl10	5.38
Cxcl14	4.28
Cytip	7.36
F2rl1	4.71
Fgr	6.65
Fyb	3.33
Gabrb2	262.3
Gfra3	3.66
Glipr1	4.39
Gpnmb	54.47
Gpr35	4.78
Gpr65	7.06
Havcr2	5.34
Hck	7.23
Hcls1	4.08
Ifit1	4.68
Itga10	4.75
Kcnj10	5.1
Kcnn4	12.25
Klrd1	13.12
Ltbp2	4.88
Ly86	4.12
Mcm6	3.16
Ms4a4c	7.17
Myh1	11.35
Neto2	5.19
Oasl2	3.39
Pkib	3.3
Ptpn22	4.54
Ptprz1	3.09
Sash3	5.12
Selplg	3.48
Siglec5	4.15
Slamf8	6.38
Slc14a1	9.13

Slc15a3	3.6		Slc15a3	5.49
Steap1	3.21		Steap1	19.23
Tnc	4.79		Tnc	7.58
Vav1	3.02		Vav1	8.51

Down-regulated in Both (> 3 fold, FDR q < 0.05)

Neurofibroma vs nerve			Day7 after nerve injury	
**Mouse**	**FC**		**Rat**	**FC**
Acvr1c	0.13		Acvr1c	0.14
Adh1	0.32		Adh1	0.03
Adipoq	0.05		Adipoq	0.16
Alb	0.27		Alb	0.16
Aqp7	0.22		Aqp7	0.23
Car3	0.05		Car3	0.17
Car4	0.14		Car4	0.2
Cdo1	0.17		Cdo1	0.2
Cidec	0.07		Cidec	0.07
Cldn22	0.17		Cldn22	0.29
Cntf	0.3		Cntf	0.18
Col23a1	0.22		Col23a1	0.09
Cryaa	0.23		Cryaa	0.19
Crybb1	0.25		Crybb1	0.1
Cyb5r2	0.25		Cyb5r2	0.17
Cyp2e1	0.05		Cyp2e1	0.22
Ddah1	0.22		Ddah1	0.17
Ddn	0.26		Ddn	0.13
Dpep1	0.25		Dpep1	0.08
Drp2	0.26		Drp2	0.15
Eln	0.33		Eln	0.18
Fa2h	0.19		Fa2h	0.11
Fgf4	0.09		Fgf4	0.09
Fgf7	0.24		Fgf7	0.3
Filip1	0.17		Filip1	0.31
Fmod	0.16		Fmod	0.19
Gdf10	0.17		Gdf10	0.22
Gjb1	0.15		Gjb1	0.16
Gpm6a	0.31		Gpm6a	0.21

Jakmip2	0.33
Kcnk1	0.26
Kif13b	0.24
Lect1	0.29
Lep	0.04
Lgals12	0.27
Limch1	0.3
Lyve1	0.28
Mal	0.3
Mc2r	0.2
Mettl7b	0.28
Mgll	0.23
Mrap	0.29
Myrip	0.3
Nrgn	0.28
Pck1	0.15
Pfkfb1	0.22
Pla2g5	0.08
Pllp	0.24
Prr15	0.23
Prx	0.23
Reep6	0.31
Retn	0.08
Rhobtb3	0.23
Sema4g	0.17
Sema5a	0.18
Slc15a2	0.24
Slc36a2	0.17
Slc6a4	0.31
Slc7a10	0.31
St8sia5	0.15
Sult1a1	0.32
Tagln	0.19
Tesk2	0.28
Thrsp	0.3
Tmem40	0.21
Wisp1	0.32

Jakmip2	0.15
Kcnk1	0.11
Kif13b	0.31
Lect1	0.09
Lep	0.09
Lgals12	0.16
Limch1	0.18
Lyve1	0.22
Mal	0.22
Mc2r	0.12
Mettl7b	0.33
Mgll	0.23
Mrap	0.11
Myrip	0.21
Nrgn	0.24
Pck1	0.09
Pfkfb1	0.31
Pla2g5	0.14
Pllp	0.27
Prr15	0.18
Prx	0.25
Reep6	0.15
Retn	0.3
Rhobtb3	0.28
Sema4g	0.19
Sema5a	0.1
Slc15a2	0.21
Slc36a2	0.13
Slc6a4	0.17
Slc7a10	0.29
St8sia5	0.08
Sult1a1	0.11
Tagln	0.33
Tesk2	0.28
Thrsp	0.11
Tmem40	0.18
Wisp1	0.28

Up-regulated in Both (> 3 fold, FDR q < 0.05)

Neurofibroma vs nerve	
**Mouse**	**FC**
Aif1	4.46
Ajap1	3.04
Apobec1	3.37
Bdnf	5.73
Btc	5.86
Ccl12	3.81
Ccl5	4.56
Cd3e	3.56
Cd72	5.04
Clec5a	3.49
Coro1a	3.53
Cst6	3.46
Cxcl10	4.58
Cytip	3.33
Fgr	3.82
Fyb	3.41
Gabrb2	4.54
Gfra3	9.8
Glipr1	3.11
Gpnmb	3.97
Gpr35	6.99
Gpr65	3.31
Havcr2	4.14
Hck	4.73
Hcls1	3.19
Ifit1	4.41
Itga10	3.01
Kcnn4	7.12
Klrd1	4.46
Ly86	6.14
Ms4a4c	5.59
Myh1	5.07
Oasl2	4.28
Pkib	6.2
Ptpn22	3.12

Day14 after nerve injury	
**Rat**	**FC**
Aif1	3.93
Ajap1	5.68
Apobec1	6.3
Bdnf	5.12
Btc	7.26
Ccl12	4355.37
Ccl5	3.15
Cd3e	9.52
Cd72	3.44
Clec5a	4.14
Coro1a	4.45
Cst6	59.82
Cxcl10	7.85
Cytip	7.78
Fgr	5.81
Fyb	3.2
Gabrb2	522.45
Gfra3	4.81
Glipr1	4.38
Gpnmb	70.92
Gpr35	5.52
Gpr65	8.91
Havcr2	5
Hck	7.24
Hcls1	4.21
Ifit1	6.31
Itga10	3.15
Kcnn4	9.4
Klrd1	19.39
Ly86	4.73
Ms4a4c	10.66
Myh1	9.91
Oasl2	7.71
Pkib	4.7
Ptpn22	4.22

Sash3	3.21
Selplg	3.43
Siglec5	7.02
Slamf8	3.51
Slc14a1	5.65
Slc15a3	3.6
Slitrk6	5.43
Steap1	3.21
Tnc	4.79
Vav1	3.02

Sash3	5.33
Selplg	4.36
Siglec5	3.23
Slamf8	6.33
Slc14a1	7.36
Slc15a3	6.45
Slitrk6	4.25
Steap1	14.87
Tnc	5.39
Vav1	6.95

Down-regulated in Both (> 3 fold, FDR q < 0.05)

**Neurofibroma vs nerve**	
**Mouse**	**FC**
Acvr1c	0.13
Adh1	0.32
Adipoq	0.05
Alb	0.27
Aqp7	0.22
Car3	0.05
Car4	0.14
Cdo1	0.17
Cidec	0.07
Cntf	0.3
Col23a1	0.22
Cryaa	0.23
Crybb1	0.25
Cyb5r2	0.25
Cyp2e1	0.05
Ddah1	0.22
Ddn	0.26
Dpep1	0.25
Drp2	0.26
Eln	0.33
Fa2h	0.19
Fgf4	0.09
Fmod	0.16

**Day14 after nerve injury**	
**Rat**	**FC**
Acvr1c	0.18
Adh1	0.12
Adipoq	0.31
Alb	0.01
Aqp7	0.31
Car3	0.3
Car4	0.15
Cdo1	0.28
Cidec	0.17
Cntf	0.17
Col23a1	0.05
Cryaa	0.16
Crybb1	0.09
Cyb5r2	0.22
Cyp2e1	0.17
Ddah1	0.2
Ddn	0.29
Dpep1	0.09
Drp2	0.2
Eln	0.31
Fa2h	0.21
Fgf4	0.07
Fmod	0.33

Gsta3	0.2
Jakmip2	0.33
Kif13b	0.24
Lect1	0.29
Lep	0.04
Lgals12	0.27
Limch1	0.3
Lyve1	0.28
Mc2r	0.2
Mettl7b	0.28
Mrap	0.29
Myrip	0.3
Nrgn	0.28
Pck1	0.15
Pfkfb1	0.22
Pla2g5	0.08
Prr15	0.23
Reep6	0.31
Sema5a	0.18
Slc15a2	0.24
Slc36a2	0.17
Smtn	0.25
St8sia5	0.15
Sult1a1	0.32
Tagln	0.19
Tesk2	0.28
Thrsp	0.3
Tmem27	0.17
Wisp1	0.32

Gsta3	0.01
Jakmip2	0.07
Kif13b	0.31
Lect1	0.08
Lep	0.15
Lgals12	0.19
Limch1	0.22
Lyve1	0.22
Mc2r	0.09
Mettl7b	0.32
Mrap	0.21
Myrip	0.31
Nrgn	0.3
Pck1	0.3
Pfkfb1	0.31
Pla2g5	0.09
Prr15	0.21
Reep6	0.2
Sema5a	0.19
Slc15a2	0.25
Slc36a2	0.33
Smtn	0.31
St8sia5	0.21
Sult1a1	0.19
Tagln	0.28
Tesk2	0.3
Thrsp	0.2
Tmem27	0.01
Wisp1	0.31
